# Efficacy and Safety of Tirzepatide Versus Dulaglutide in Type 2 Diabetes With or Without Established Atherosclerotic Cardiovascular Disease: A Network Meta‐Analysis of Randomized Clinical Trials

**DOI:** 10.1002/edm2.70313

**Published:** 2026-09-07

**Authors:** Ahmed W. Hageen, Ahmed Farid Gadelmawla, Ahmad Omar Saleh, Salma Bahnasy, Safir Eladawi, Moatasem Abdelaziz, Khalil Iyad, Ahmed Hamdy Kandil, Karim Zinhom, Mohamed Reyad Mohamed, Hind Abdulhay, Mustafa Turkman, Basel Abdelazeem, Gregg C. Fonarow

**Affiliations:** ^1^ Faculty of Medicine Tanta University Tanta Egypt; ^2^ Faculty of Medicine Menoufia University Menoufia Egypt; ^3^ Medical Research Group of Egypt (MRGE) Negida Academy Arlington Massachusetts USA; ^4^ Faculty of Medicine The University of Jordan Amman Jordan; ^5^ Matruh Specialized Center for Cardiac Surgery and Interventional Catheter Marsa Matruh Egypt; ^6^ Faculty of Medicine Ain Shams University Cairo Egypt; ^7^ Faculty of Medicine Kafr Elsheikh University Kafr Elsheikh Egypt; ^8^ Faculty of Medicine Alexandria University Alexandria Egypt; ^9^ Faculty of Medicine Benha University Benha Egypt; ^10^ Faculty of Medicine October 6 University Giza Egypt; ^11^ Internal Medicine Department University of Arizona College of Medicine–Phoenix Phoenix Arizona USA; ^12^ Faculty of Medicine Mansoura University Mansoura Egypt; ^13^ Faculty of Medicine Michigan State University East Lansing Michigan USA; ^14^ Division of Pulmonary and Critical Care University of Toledo Toledo Ohio USA; ^15^ Department of Cardiology West Virginia University Morgantown West Virginia USA; ^16^ Division of Cardiology University of California Los Angeles Los Angeles California USA

**Keywords:** cardiometabolic outcomes, dulaglutide, RCTs, Tirzepatide, type 2 diabetes mellitus

## Abstract

**Background and Aim:**

This network meta‐analysis addresses the limited dose‐specific comparative evidence by evaluating different doses of tirzepatide (TZP) versus dulaglutide (DULA) for glycemic control, weight loss, and safety in type 2 diabetes mellitus (T2DM).

**Methods:**

Following PRISMA and Cochrane guidance, we searched major databases for RCTs comparing TZP and DULA in adults with T2DM. Efficacy outcomes included body weight change (Weeks 12 and 16) and mean HbA1c change (Weeks 12 and 24). Safety outcomes included mortality, cardiovascular events, and adverse events (AEs). SUCRA ranking was used and analysis performed in RStudio (v4.5.1).

**Results:**

Four RCTs comprising 14,348 participants contributed to a seven‐node network. However, Nicholls et al. 2025 had the majority of sample size (*n* = 13,165). TZP 15 mg achieved the greatest reduction in body weight at Week 16 (mean difference [MD] vs. TZP 5 mg: −2.68 kg; 95% CI −4.98 to −0.38), while TZP 1 mg (MD: 4.39 kg; 95% CI 1.28 to 7.51) and DULA 0.75 mg (MD: 3.10 kg; 95% CI 0.25 to 5.96) were associated with smaller reductions relative to TZP 5 mg. TZP 15 mg also produced the largest HbA1c reduction at Week 24 (MD vs. TZP 5 mg: −0.40%; 95% CI −0.62 to −0.19), compared with TZP 10 mg (MD: −0.23%; 95% CI −0.44 to −0.02) and DULA 1.5 mg (MD: 0.67%; 95% CI 0.25 to 1.09). No significant differences were observed between treatments for all‐cause mortality or major cardiovascular events. Gastrointestinal AEs were more frequent with higher TZP doses, especially TZP 15 mg vs. TZP 5 mg for nausea, while serious AEs and severe hypoglycemia were similar across doses.

**Conclusion:**

Higher TZP doses potentially improve efficacy, while lower TZP/DULA doses enhance tolerability, supporting individualized T2DM care. However, these findings should be interpreted cautiously because several comparisons are indirect, many participants had not reached their maintenance dose, several safety outcomes were based on limited event counts, and significant inconsistency was observed for early body‐weight outcomes.

## Graphical abstract

This network meta‐analysis of 4 randomized controlled trials (14,348 adults with type 2 diabetes, with or without established atherosclerotic cardiovascular disease) compared all clinically relevant doses of tirzepatide (1, 5, 10, 15 mg) against dulaglutide (0.75, 1.5, 4.5 mg). Tirzepatide 15 mg produced the greatest reductions in body weight at Week 16 (−2.68 kg vs. TZP 5 mg) and in HbA1c at Week 24 (−0.40%), with a clear dose–response gradient across both outcomes. All‐cause mortality and major adverse cardiovascular events did not differ significantly between treatments, while gastrointestinal adverse events increased with higher tirzepatide doses. These findings suggest higher tirzepatide doses maximize glycemic and weight‐loss efficacy, whereas lower tirzepatide/dulaglutide doses favour tolerability. These findings support individualized dose selection in type 2 diabetes management.

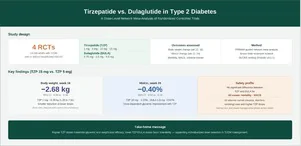

## 1 Introduction

Type 2 diabetes mellitus (T2DM) remains a substantial worldwide health burden [1]. In 2024, about 589 million adults were estimated to have diabetes mellitus (DM) globally, and this number is expected to increase to 853 million by 2050 [2]. Its impact extends beyond elevated glucose levels, as obesity and cardiovascular disease contribute importantly to complications and mortality [3]. Accordingly, contemporary management focuses on sustained reductions in haemoglobin A1c (HbA1c) while also achieving meaningful weight loss [4].

Glucagon‐like peptide‐1 receptor agonists (GLP‐1 RAs) are established options for T2DM because they improve glycemic control with a low intrinsic risk of hypoglycemia and promote weight reduction [5]. Dulaglutide (DULA) is a once‐weekly GLP‐1 RA with cardiovascular outcome evidence [6]. In the REWIND trial, DULA reduced major adverse cardiovascular events (MACE) versus placebo in people with T2DM [6]. These data support DULA as a relevant active comparator when evaluating newer incretin‐based therapies [6].

Tirzepatide (TZP) is a once‐weekly dual agonist of the glucose‐dependent insulinotropic polypeptide (GIP) and GLP‐1 receptors [7]. Early randomized evidence showed dose‐related improvements in HbA1c and body weight across multiple TZP doses, with gastrointestinal adverse events as the most frequent tolerability limitation [8]. In a phase 3 trial in Japan, TZP 5, 10, and 15 mg achieved greater reductions in HbA1c and body weight than DULA 0.75 mg [9]. A pragmatic strategy trial also compared escalating DULA to higher doses versus switching to TZP with titration to higher doses, addressing a common real‐world decision in patients inadequately controlled on submaximal DULA [10]. Finally, a cardiovascular outcomes trial compared TZP up to 15 mg with DULA 1.5 mg in patients with established atherosclerotic cardiovascular disease and demonstrated noninferiority for MACE [11]. Previous evidence reported that TZP 5, 10, and 15 mg are associated with greater reductions in HbA1c and body weight compared with DULA and other GLP‐1 RAs, but individual head‐to‐head comparisons of all dose pairs are lacking [7, 12, 13]. Although TZP (5, 10, and 15 mg) has demonstrated superior reductions in HbA1c and body weight compared with DULA and other GLP‐1 RAs, no trial simultaneously compared all clinically relevant TZP doses (1, 5, 10, 15 mg) against all approved DULA doses (0.75, 1.5, 4.5 mg), and none provided comprehensive dose‐level comparative ranking across glycemic, weight, and safety outcomes within a unified framework. However, the recently published SURPASS‐CVOT trial provided important cardiovascular outcomes [11], but did not provide comprehensive efficacy comparisons across all TZP doses for both HbA1c reduction and body weight change. A previous network meta‐analysis (NMA) lacks clear dose‐to‐dose contrast estimates of TZP and DULA [12], limiting clinicians to select the optimal dosing strategies to manage glycemic and weight outcomes. Notably, NMA can combine direct and indirect evidence across a connected treatment network to support comparative estimation when head‐to‐head evidence is limited. Therefore, we conducted a NMA of RCTs to compare different maximal tolerated doses of TZP and DULA for efficacy and safety outcomes among adults with T2DM, with or without established atherosclerotic cardiovascular disease (ASCD).

## 2 Methods

### 2.1 Data Sources and Search Strategy

This NMA was conducted following the Cochrane Handbook for Systematic Reviews of Interventions (Chapter 11: Undertaking network meta‐analyses) [14] and adhered to the PRISMA guidelines [15]. The protocol for this review was registered at PROSPERO with (ID: CRD420261293548). All prespecified outcomes and analyses described in the study protocol were reported, and no deviations from the registered protocol or analysis plan occurred. The ethical approval and institutional review board were waived because the authors relied on the data from published trials. We searched on Cochrane, Scopus, PubMed, and Web of Science through 31 December 2025 without any automated filters or language restrictions with the following search terms (Type II diabetes mellitus) OR (T2DM) OR (Diabetes mellitus type 2) OR (Diabetes type 2) AND (Tirzepatide) OR (LY‐3298176) OR (Mounjaro) OR (Zepbound) AND (Dulaglutide) OR (LY‐2189265) to retrieve the available RCTs that assess the efficacy and safety of TZP and DULA for various dose regimens in patients with T2DM. The detailed search strategy with records of each database is shown in Table S1, and the PRISMA checklist is summarized in Table S2. Additionally, a manual search was applied to the references and citations of included trials to detect any studies that were missed during the primary search.

### 2.2 Selection Criteria and Data Extraction

After retrieving the records from databases, two independent authors (S.B. and A.W.H) screened the records using Covidence software [16] in two phases: title and abstract screening, followed by full‐text screening. Conflicts were resolved by consensus and discussion with a third author (A.F.G). Studies were eligible if they met the following PICOS framework: Population (P): Adults with T2DM, regardless of prior antihyperglycemic treatment status (treatment‐naïve, on oral monotherapy, or on submaximal GLP‐1 receptor agonist therapy), with or without established ASCD. Intervention (I): Tirzepatide (dual GIP/GLP‐1 receptor agonist), any once‐weekly subcutaneous dose (1–15 mg), administered either as initiation therapy or as a switch from another GLP‐1 RA. Comparator (C): Dulaglutide (selective GLP‐1 receptor agonist), any once‐weekly approved or protocol‐specified dose (0.75–4.5 mg), as active comparator. Outcomes (O): Efficacy outcomes included body weight (kg) change from baseline (Week 12), body weight (kg) change from baseline (Week 16), mean change in HbA1c (%) from baseline (Week 12), and mean change in HbA1c (%) from baseline (Week 24). Safety outcomes included any adverse event (AE) during treatment, nausea, diarrhoea, vomiting, all‐cause death, severe hypoglycemia, serious adverse events (Serious AE), headache, injection site reaction, dyspepsia, constipation, pancreatitis, adverse events leading to drug discontinuation, and MACE (composite of cardiovascular death, myocardial infarction, or stroke). We excluded the non‐randomized studies, non‐human or in vitro studies, reviews, meta‐analyses, editorials, guidelines, conference abstracts, book chapters, and duplicate publications or studies with overlapping patient cohorts. The other four independent authors (M.A., K.I., A.H.K., and M.R.M) extracted the study features, baseline characteristics (e.g., age; sex; body weight, body mass index (BMI), and waist circumference (WC); race/ethnicity; cardiovascular parameters (systolic and diastolic blood pressure and heart rate); fasting serum glucose; HbA1c; eGFR; lipid profile; concomitant medications; and duration of T2DM). Additionally, the efficacy and safety outcomes, as mentioned in the PICOS format. Any discrepancies were resolved through consensus with a third reviewer (A.W.H.) through a discussion.

### 2.3 Definitions of MACE Outcome

The primary outcome of SURPASS‐CVOT was three‐point MACE (cardiovascular death, non‐fatal myocardial infarction [MI] or non‐fatal stroke); secondary outcome was 4‐point MACE (3‐point MACE or coronary revascularization). SURPASS‐SWITCH and SURPASS J‐mono reported adjudicated MACE, but did not provide any definitions of the individual components. Frias et al. [8] employed a composite endpoint of cardiovascular or non‐cardiovascular death, MI, stroke, transient ischemic attack, supraventricular arrhythmia, and coronary revascularization. The common 3‐point MACE definition was used as much as possible in the pooled analyses. Each trial adjudicated and analyzed MACE components (MI and stroke).

### 2.4 Assessment of the Quality of Included Studies

The Cochrane risk of bias assessment tool (ROB 2.0) [17] was used by two independent authors (M.R.M. and A.W.H.) to assess the quality of included RCTs. We assessed risk of bias across five domains: deviations from intended interventions, missing outcome data, randomization process, outcome measurement, and selective reporting. The RCTs were assessed by three decisions: high risk, some concerns, and low risk of bias.

### 2.5 Statistical Methods

#### 2.5.1 The Geometry of the Network

We presented a network graph with seven nodes representing different doses of TZP and DULA. The edges represent available direct comparisons between pairs of routes. The line thickness indicates the number of studies involved in these comparisons. The plotted number represents the number of comparisons between each node.

### 2.6 Data Synthesis and Analysis

The frequentist method was employed to conduct NMA of the included studies [18]. Comparing different doses of TZP and DULA. Random effects models were used in our research to express treatment effects as relative risk (RR) and mean differences (MD) with corresponding 95% confidence intervals (95% CI). We calculated the surface under the cumulative ranking curve (SUCRA) for each outcome. The relative ranking of the different doses to determine efficacy and safety was estimated based on the distribution of the SUCRAs. Higher SUCRA values indicated better performance. We utilized the *I* ^2^ statistic to assess heterogeneity in the network analysis. Inconsistency in the model was evaluated by comparing estimates from direct and indirect comparisons. We compared the distributions of key study characteristics across studies grouped by contrast to evaluate transitivity. Statistical inconsistencies between direct and indirect evidence were examined using a combination of global and local approaches. Furthermore, we applied a design‐by‐treatment interaction model to investigate inconsistencies from all sources in the entire network [19, 20]. Local inconsistency was evaluated based on the node‐splitting method [21]. Statistical analyses were conducted using the “netmeta” package in R (v4.5.1) using RStudio [22, 23]. A *p*‐value of less than 0.05 was considered statistically significant. For trials that did not report numerical outcome data, values were extracted from the figures using a validated graphical extraction tool (WebPlotDigitizer) [24]. The digitization strategy was carried out independently by two reviewers, who independently calibrated axes and manually digitized the data points for weight‐related and glycemic outcomes at every follow‐up reported. If there were disagreements, a consensus was established during discussion. Furthermore, and when necessary, we have used the established statistical method (Meta Accelerator web tool) for the extraction of mean and standard deviation values, which are thus converged [25]. In particular, summary statistics reported were converted to approximate means and standard deviations so as to be able to be included in the meta‐analysis with consistency. These measures allowed for comparable outcome measures across studies and for convergence in the pooled analysis. For HbA1c, Weeks 12 and 24 were selected, and for body weight, Weeks 12 and 16 were selected as the common time points when the efficacy was assessed across the trials included in this analysis, which enabled the building of a coherent and comparable treatment network. The outcome of Week 12 and Week 24 of Frias et al. shows mainly intermediate target‐dose exposure, as a result of the dose escalation protocol. Besides, to assess the credibility of our findings and, a sensitivity analysis was performed after excluding Nicholls et al. [11], which involved the largest number of patients compared to other included trials and also has longer diabetes duration and established ASCD. Besides, TZP was administered as a titrated dose (up to 15 mg or maximum tolerated dose) in Nicholls et al. [11]. The sensitivity analysis was performed by repeating all NMA after excluding this study [11] from outcomes to which it contributed data. All‐cause mortality, MACE, overall AE, serious AE, treatment discontinuation due to AE, severe hypoglycemia, nausea, vomiting, diarrhoea, and pancreatitis were all included as outcomes. Body‐weight change, HbA1c reduction, and various other AE outcomes, including headache, abdominal distention, injection‐site reactions, hyperglycemia, dyspepsia, constipation, and gastrointestinal disorders resulting in treatment discontinuation, were not outcomes for which the largest trial [11] provided data. This sensitivity analysis did not have an impact on these outcomes. The results of the sensitivity analyses were compared with the primary analyses to explore the robustness of the network estimates and treatment rankings. Additional sensitivity analyses were conducted: (1) excluding the SURPASS‐SWITCH trial because it was open‐label and used DULA 4.5 mg comparator arm; (2) excluding the SURPASS J‐mono trial [9] because it had DULA 0.75 mg (the approved dose in Japan) comparator arm instead of the 1.5 mg standard used in other trials; (3) restricting pooled HbA1c and body weight analyses to treatment‐naïve populations by excluding the GLP‐1 RA–experienced switch population from the SURPASS‐SWITCH trial.

## 3 Results

### 3.1 Search Results and Study Selection

**FIGURE 1:**
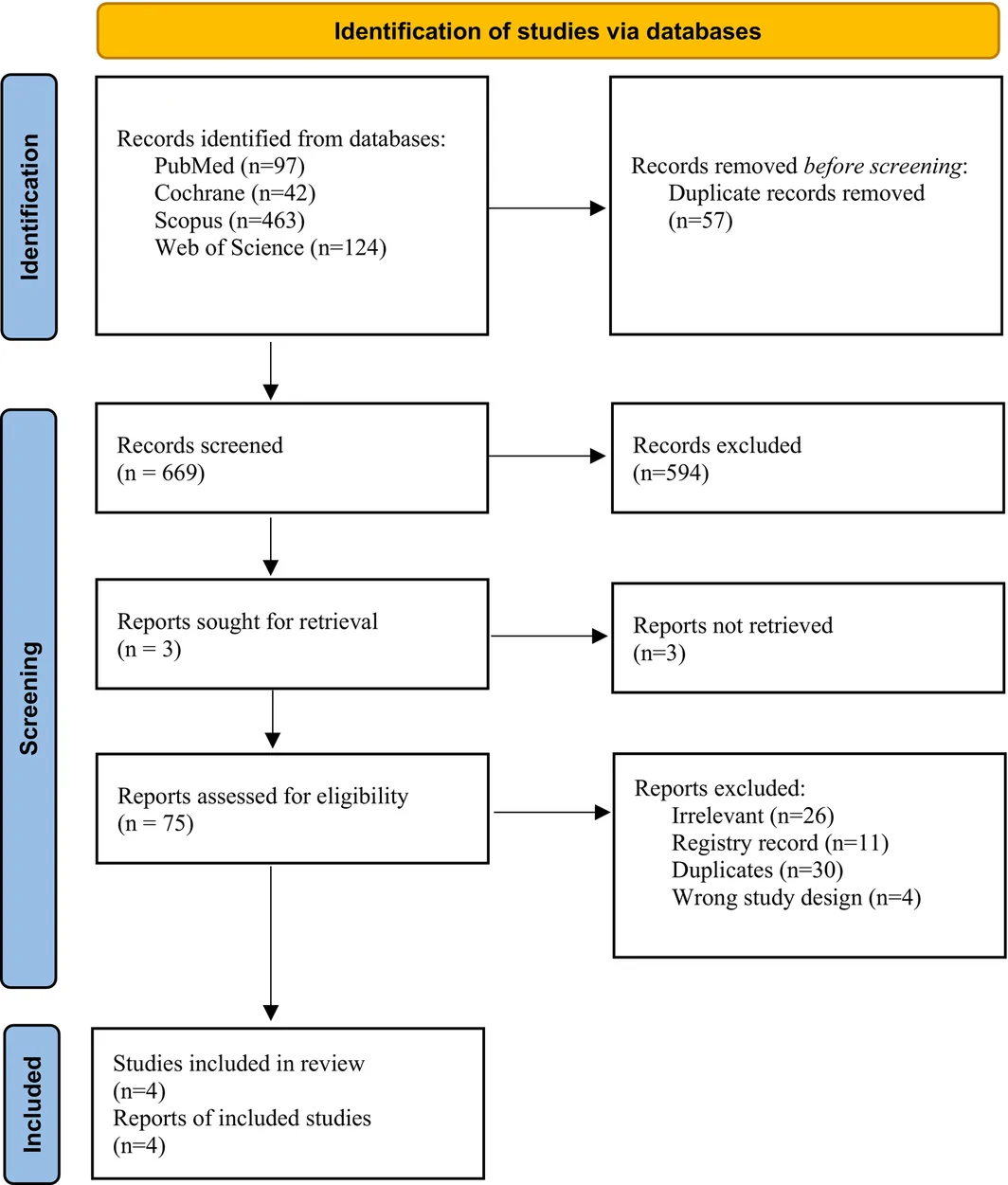
PRISMA flow chart of the screening process.

An electronic search across four databases resulted in 726 references. 57 references were removed after Covidence identified duplications; no duplicate references were removed manually. From a total of 669 references, 594 references were excluded after screening the title and abstract. A total of 75 references were retained for full‐text screening; however, 71 studies were excluded after full‐text screening, and four studies met our eligibility criteria (Figure 1).

### 3.2 Characteristics of the Included Studies

Four RCTs [8, 9, 10, 11] included 14,348 patients in our data synthesis; however, [11] had the majority of sample size (*n* = 13,165). This large sample size was addressed by conducting a sensitivity analysis for applicable outcomes for analysis. A summary of characteristics of included trials is presented in Table 1A, and baseline characteristics of participants are presented in Table 1B. The TZP group consisted of 7413 patients, while the DULA group consisted of 6935 patients. The mean age of patients in the TZP group was 57.6 years, ranging from 56.0 to 64.0 years. However, the mean age for each TZP dose (1, 5, 10, and 15 mg) was 57.4, 57.4, 56.4, and 58.5 years, respectively. Additionally, the mean age for each DULA dose (0.75, 1.5, and 4.5 mg) was 57.5, 61.4, and 57.3 years, respectively. Besides, the DULA group had a mean age of 59.4 years, ranging from 57.3 to 64.1 years.

**TABLE 1A:** Summary of included studies.

Study ID	Design, centres	Trial registry ID	Region (s)	Control details	Intervention details	Inclusion criteria	Outcomes	Doses	Sample size	Follow‐up period	Main conclusion
Nicholls et al. 2025 [11]	RCT, multicentre	NCT04255433	Argentina, Australia, Austria, Belgium, Brazil, Canada, China, Czech Republic, France, Germany, Greece, Hungary, India, Israel, Italy, Japan, Mexico, Netherlands, Poland, Romania, Russia, Slovakia, South Korea, Spain, Sweden, Taiwan, Turkey, Ukraine, UK, and the USA	DULA once weekly	S.C.TZP once weekly, and escalated from 2.5 mg up to a maximum of 15 mg or the MTD	Adults aged ≥ 40 years with T2DM. HbA1c 7.0%–10.5% (53–91 mmol/mol). BMI ≥ 25 kg/m^2^. Established ASCVD in at least one vascular territory (coronary, cerebrovascular, or peripheral arterial disease). Provision of written informed consent.	• A composite of death from cardiovascular causes, MI, or stroke, assessed in a time‐to‐first‐event analysis. • AE‐related to the treatment	• TZP: Initiated at 2.5 mg and escalated by 2.5 mg increments every 4 weeks up to a maximum dose of 15 mg. • DULA: Maintained at a dose of 1.5 mg throughout the trial	13,165	Median duration of 4 years.	TZP was noninferior to DULA with respect to the composite endpoint of death from cardiovascular causes, MI, or stroke in T2DM and ASCD
Billings et al. 2025 [10]	RCT, multicentre	NCT05564039	USA, Mexico, Belgium, Germany, and Romania	DULA escalated every 4 weeks	S.C. DULA to S.C. TZP. at a dose of 2.5 mg once weekly and was escalated in 2.5‐mg increments every 4 weeks until a dose of 15 mg or the MTD was achieved	Adults (≥ 18 years) with TADM, HbA1c ≥ 7.0% to ≤ 9.5%, stable body weight, and BMI ≥ 25 kg/m^2^. They must have been receiving a stable dose of DULA (0.75 or 1.5 mg) for at least 6 months and 0–3 oral anti‐hyperglycemic medications for at least 3 months prior to the study	• Change from baseline in HbA1c at week 40. • AE‐related to the treatment	• TZP: 2.5, 5, 7.5, 10, 12.5, and 15 mg (target dose: 15 mg or MTD). • DULA: 0.75, 1.5, 3.0, and 4.5 (target dose: 4.5 mg or MTD)	282	40‐week treatment period followed by a 4‐week safety follow‐up period	In adult with T2DM inadequately controlled on submaximal doses of DULA, switching to TZP resulted in significantly greater HbA1c reduction and weight loss compared with escalating the dose of DULA
Inagaki et al. 2022 [9]	RCT, multicentre	NCT03861052	Japan	DULA once weekly	S.C. TZP once weekly	Adults aged ≥ 20 years with T2DM. Either treatment‐naïve (HbA1c 7.0%–10.0%) or on oral anti‐hyperglycemic monotherapy (HbA1c 6.5%–9.0% at Visit 1, 7.0%–10.0% at Visit 2). Willing to wash out prior medication for 8 weeks if applicable. Stable weight (change ≤ 5%) in the 3 months prior. BMI ≥ 23 kg/m^2^ at screening	• Mean change in HbA1c from baseline at week 52 • AE‐related to the treatment	• TZP: Administered at doses of 5 mg, 10 mg, or 15 mg. • DULA: 0.75 mg once per week.	636	52‐week treatment period followed by a 4‐week safety follow‐up period.	TZP was superior to DULA for glycaemic control and reduction in bodyweight, with a safety profile consistent with that of GLP‐1 receptor agonists, indicating a potential therapeutic use in patients with T2DM
Frias et al. 2018 [8]	RCT, multicentre	NCT03131687	Poland, Puerto Rico, Slovakia, and the USA	DULA once weekly	S.C. TZP once weekly	Adults aged 18–75 years with T2DM for ≥ 6 months. HbA1c 7.0%–10.5% (inclusive). Inadequate control with diet and exercise alone or with stable metformin therapy for ≥ 3 months. BMI 23–50 kg/m^2^	• Change in HbA1c from baseline to 26 weeks in the modified intention‐to‐treat population • AE‐related to the treatment	• TZP: 1, 5, 10, or 15 mg. (The 10 and 15 mg doses were achieved via titration starting at 5 mg). • DULA: 1.5 mg.	316	26 weeks for treatment and 4 weeks followed‐up for safety outcomes	TZP showed significantly better efficacy with regard to glucose control and weight loss than DULA, with an acceptable safety and tolerability profile.

Abbreviations: AE, adverse events; ASCD, atherosclerotic cardiovascular disease; BMI, body mass index; DULA, dulaglutide; HA1c, haemoglobin; MI, myocardial infarction; MTD, maximum tolerated dose; RCT, randomized controlled trial; S.C, subcutaneous; T2DM, type 2 diabetes mellitus; TZP, tirzepatide.

**TABLE 1B:** Baseline characteristics of the included studies^a^.

Study ID	Groups	Number of participants	Age— yr.	Male, *n* (%)	Weight, kg	BMI, kg/m^2^	WC, cm	Race/Ethnicity, *n* (%)			Cardiovascular parameters			FSG level, mg/dL	HbA1c,%	Lipid level—mg/dl					Duration of T2DM — yr.
								Asian	H/L	White	BP— mm Hg		HR—beats/min			Triglycerides	Total cholesterol	LDL‐C	VLDL‐C	HDL‐C	
											SBP	DBP									
Nicholls et al. 2025 [11]^b^	TZP 15 mg	6586	64 ± 8.8	4695 (71.3)	92.6 ± 18.9	32.6 ± 5.5	N/A	N/A	1988 (30.2)	5299 (81.5)	135.1 ± 15.5	77.9 ± 9.7	N/A	N/A	8.40 ± 0.92	166.2 ± 89.02	N/A	80.5 ± 36.8	N/A	N/A	14.8 ± 8.8
	DULA 1.5 mg	6579	64.1 ± 8.7	4653 (70.7)	92.5 ± 18.8	32.6 ± 5.5	N/A	N/A	1981 (30.1)	5282 (81.4)	135.5 ± 15.8	78.1 ± 9.7	N/A	N/A	8.38 ± 0.93	165.2 ± 88.98	N/A	80.7 ± 38.0	N/A	N/A	14.7 ± 8.7
Billings et al. 2025 [10]	TZP 15 mg	139	57.9 ± 9.8	75 (54.0)	98.0 ± 20.8	34.7 ± 6.2	N/A	3 (2.2)	18 (50.0)	109 (78.4)	129.9 ± 16.1	80.5 ± 9.3	N/A	150.0 ± 36.6	7.82 ± 0.69	188.3 ± 111.2	170.4 ± 46.3	92.9 ± 40.1	34.3 ± 14.3	41.7 ± 10.5	11.1 ± 6.9
	DULA 4.5 mg	143	57.3 ± 10.0	71 (49.7)	95.8 ± 20.3	34.0 ± 6.5	N/A	1 (0.7)	14 (42.4)	120 (83.9)	129.0 ± 14.5	80.3 ± 8.1	N/A	156.2 ± 45.6	7.82 ± 0.73	192.6 ± 141.8	170.9 ± 43.9	93.3 ± 41.0	32.7 ± 14.1	42.1 ± 11.5.	11.3 ± 7.1
Inagaki et al. 2022 [9]	TZP 5 mg	159	56.8 ± 10.1	113 (71)	78.5 ± 16.3	28.6 ± 5.4	97.1 ± 10.1	159 (100)	N/A	N/A	130.2 ± 12.7	82.4 ± 9.7	72.8 ± 10.8	162.0 ± 31.85	8.2 ± 0.9	150.9 ± 81.46	194.9 ± 34.8	107.8 ± 29.6	30.2 ± 16.77	51.3 ± 12.99	4.65 ± 4.00
	TZP 10 mg	158	56.2 ± 10.3	119 (75)	78.9 ± 13.7	28.0 ± 4.1	96.3 ± 10.2	158 (100)	N/A	N/A	130.0 ± 15.6	82.6 ± 10.0	72.9 ± 10.2	160 ± 33.33	8.2 ± 0.9	153.5 ± 85.73	191.0 ± 34.2	103.9 ± 28.6	30.7 ± 17.09	50.3 ± 12.8	5.2 ± 4.59
	TZP 15 mg	160	56.0 ± 10.7	132 (83)	78.9 ± 14.3	28.1 ± 4.4	96.5 ± 10.1	160 (100)	N/A	N/A	132.2 ± 13.8	83.9 ± 10.0	72.8 ± 9.7	165 ± 39.26	8.2 ± 0.9	145.5 ± 79.18	186.9 ± 32.5	103.2 ± 28.4	29.1 ± 15.8	48.7 ± 12.14	5.2 ± 4.59
	DULA 0.75 mg	159	57.5 ± 10.2	117 (74)	76.5 ± 13.2	27.8 ± 3.7	94.9 ± 10.1	159 (100)	N/A	N/A	130.6 ± 15.4	82.1 ± 10.2	73.0 ± 10.8	159.33 ± 30.37	8.2 ± 0.9	138.2 ± 75.15	190.8 ± 33.1	107.5 ± 30.0	27.6 ± 15.0	50.8 ± 12.6	4.7 ± 4.00
Frias et al. 2018 [8]	TZP 1 mg	52	57.4 ± 8.9	29 (56)	93.2 ± 24.4	32.9 ± 6.1	109.9 ± 14.7	0 (0)	25 (52)	42 (81)	126.8 ± 12.98	75.2 ± 8.22	74.6 ± 10.24	161.1 ± 40.7	8.2 ± 0.9	168.28 ± 108.59	174.02 ± 44.5	88.94 ± 36.23	34.80 ± 16.73	50.27 ± 13.94	7.8 ± 5.4
	TZP 5 mg	55	57.9 ± 8.2	34 (62)	92.8 ± 19.0	32.9 ± 5.7	110.1 ± 14.8	0 (0)	22 (49)	46 (84)	127.8 ± 12.98	76.6 ± 8.23	71.8 ± 10.23	168.6 ± 44.3	8.2 ± 1.0	194.85 ± 111.67	185.62 ± 43.02	100.54 ± 37.28	34.80 ± 14.34	50.27 ± 14.34	8.9 ± 5.7
	TZP 10 mg	51	56.5 ± 9.9	30 (59)	92.7 ± 19.5	32.6 ± 5.8	109.6 ± 14.6	1 (2)	26 (57)	37 (74)	125.8 ± 12.99	76.2 ± 8.21	73.2 ± 10.21	170.6 ± 50.3	8.2 ± 1.1	186.00 ± 94.88	181.75 ± 44.18	100.54 ± 38.66	34.80 ± 16.57	46.40 ± 13.81	7.9 ± 5.8
	TZP 15 mg	53	56.0 ± 7.6	22 (42)	89.1 ± 22.7	32.2 ± 6.2	107.6 ± 15.8	1 (2)	23 (46)	43 (81)	126.0 ± 13.03	77.1 ± 8.22	70.8 ± 10.19	164.8 ± 48.6	8.1 ± 1.1	212.57 ± 116.07	193.35 ± 45.04	112.14 ± 36.60	34.80 ± 16.89	46.40 ± 14.08	8.5 ± 6.1
	DULA 1.5 mg	54	58.7 ± 7.8	24 (44)	89.8 ± 16.9	32.4 ± 5.4	108.5 ± 14.8	2 (4)	19 (41)	44 (83)	125.7 ± 13.0	75.2 ± 8.23	73.2 ± 10.21	178.1 ± 64.5	8.1 ± 1.0	168.28 ± 110.64	189.48 42.63	104.41 ± 36.94	34.80 ± 14.21	50.27 ± 14.21	9.3 ± 7.1

Abbreviations: ASCD, atheroscelerotic cardiovascular disease; BMI, body mass index; DBP, diastolic blood pressure; DULA, dulaglutide; FSG, fasting serum glucose; H/L, Hispanic or Latino; HA1c, haemoglobin; HDL‐C, high‐density lipoprotein cholesterol; HR, heart rate; LDL‐C, low‐density lipoprotein cholesterol; N/A, not applicable; SBP, systolic blood pressure; SC, subcutaneous; T2DM, type 2 diabetes mellitus; TZP, tirzepatide; VLDL‐C, very‐low‐density lipoprotein cholesterol; WC, waist circumference.

^a^ Plus–minus values are means ±SD.

^b^ TZP was dosed per a titrate‐to‐target strategy (up to 15 mg or maximum tolerated dose).

### 3.3 Risk of Bias Assessment

**FIGURE 2:**
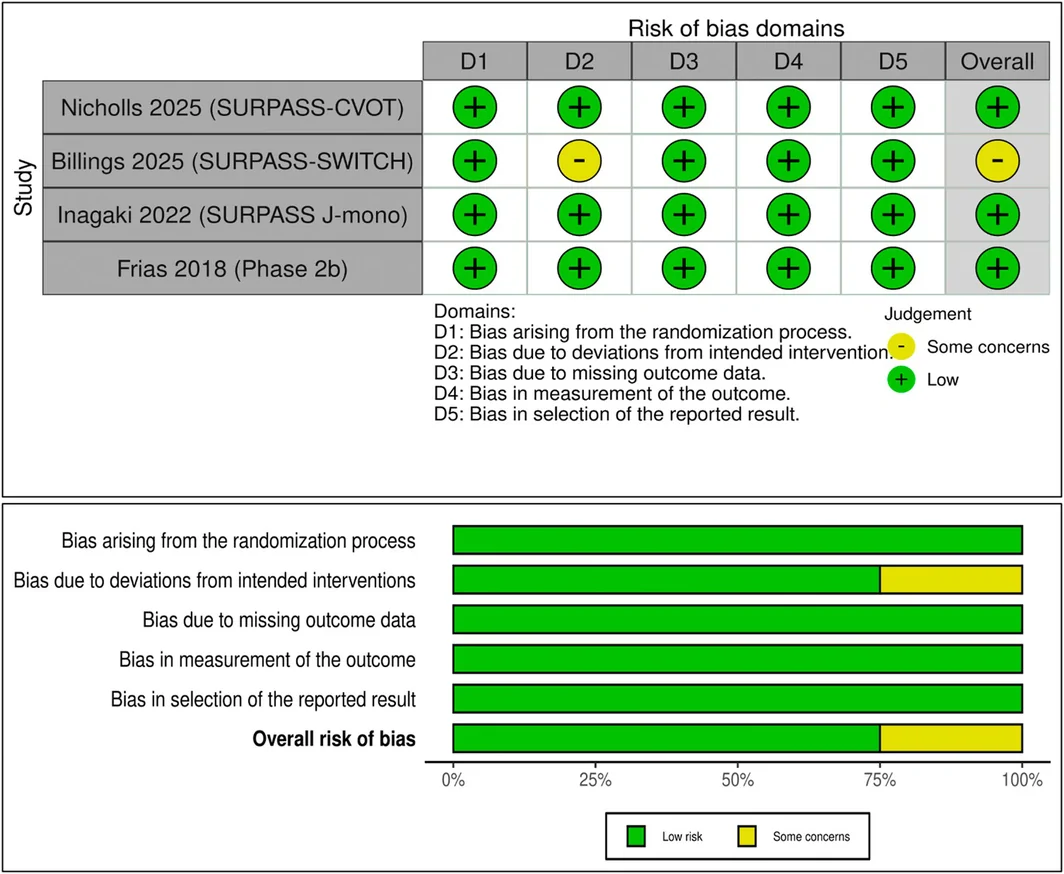
Quality assessment of risk of bias in the included trials. The upper panel presents a schematic representation of risks (low = green and some concerns = yellow) for specific types of biases of each study in the review. The lower panel presents risks (low = green and some concerns = yellow) for the subtypes of biases of the combination of studies included in this review.

Overall, three RCTs [8, 9, 11] were rated as having low risk of bias in all domains of risk of bias assessment, including bias arising from the randomization process, bias due to deviations from intended interventions, bias due to missing outcome data, bias in measurement of the outcome, and bias in selection of the reported result. However, one study was evaluated as some concerns [10] due to open‐label design, as participants and treating clinicians knew treatment assignment (Figure 2). All studies have shown a low risk due to adequate sequence generation and balanced baselines.

### 3.4 Meta‐Analysis

#### 3.4.1 Body Weight (Kg) Change From Baseline

The network meta‐analysis evaluated the effect of different doses of TZP and DULA on the body weight change from baseline at Weeks 12 and 16. At Week 12, three studies were evaluated involving 1156 participants, and seven doses were assessed. At Week 16, three studies were evaluated involving 1144 participants, and six doses were assessed.

**FIGURE 3:**
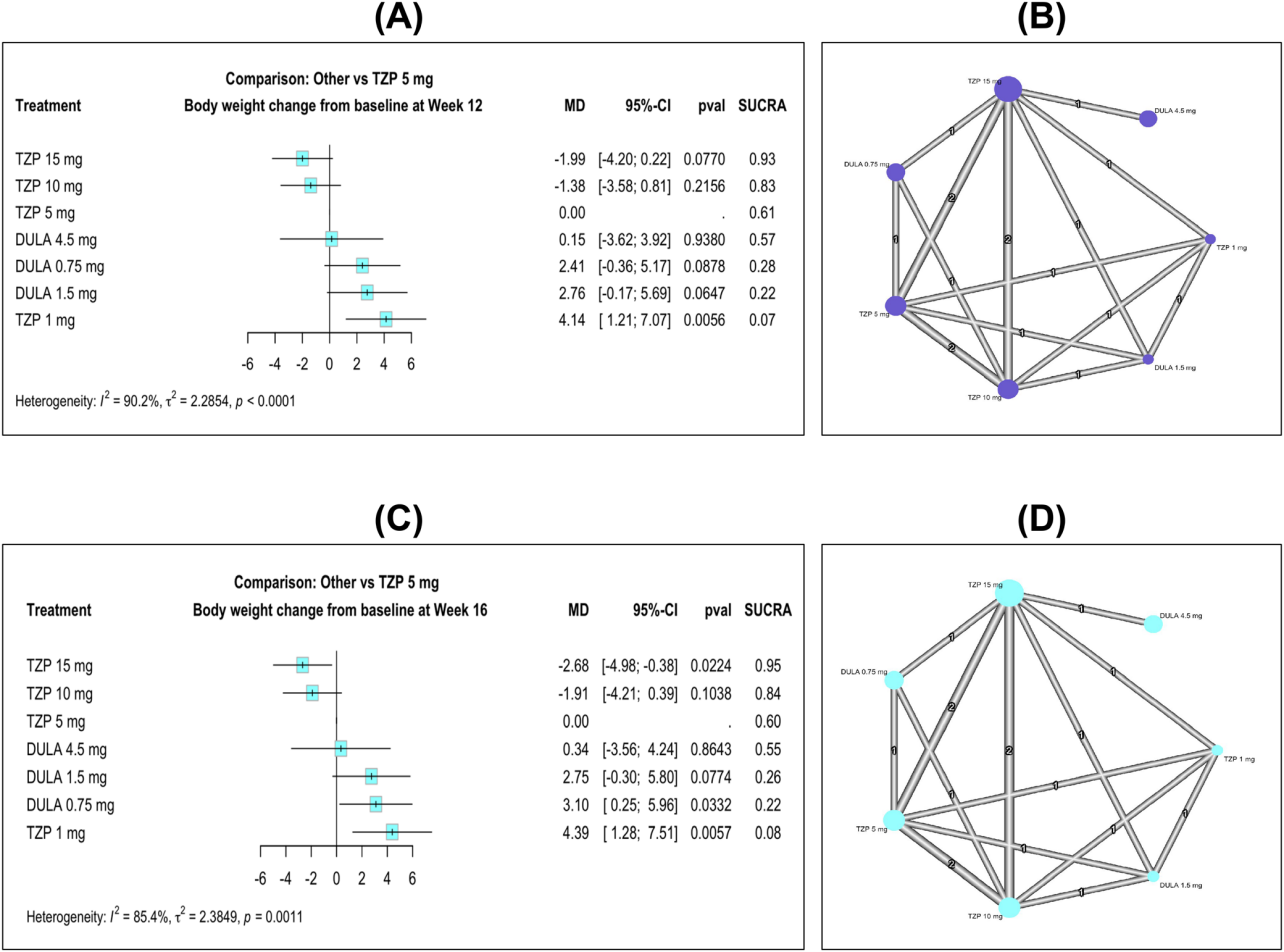
Body weight‐related outcomes in Kilogrammes (Kg): (A) Forest plot for body weight (Kg) change from baseline at week 12; (B) Net graph of network meta‐analysis for body weight (Kg) change from baseline at week 12; (C) Forest plot for body weight (Kg) change from baseline at Week 16; (D) Net graph of network meta‐analysis for body weight (Kg) change from baseline at Week 16.

At Week 12, TZP 1 mg was associated with the lowest weight change from baseline compared to 5 mg TZP (MD: 4.14, 95% CI: [1.21; 7.07], *p* = 0.0056). However, the remaining comparisons showed no significant differences compared with 5 mg TZP. Based on SUCRA, the TZP 15 mg (SUCRA: 0.93) was ranked as most effective in weight reduction, followed by TZP 10 mg (SUCRA: 0.83). In contrast, TZP 1 mg showed one of the smallest effects in weight reduction (SUCRA: 0.07) (Figure 3A). The pooled studies showed substantial heterogeneity (*I* ^2^ = 90.2%). A network graph for body weight (Kg) change from baseline at Week 12 is illustrated in Figure 3B. All pairwise comparisons of relative TZP vs. DULA effects for change in body weight (Kg) at Weeks 12 and 16 are demonstrated in Table 1C.

**TABLE 1C:** League table of network meta‐analysis displaying relative TZP vs. DULA effects for all pairwise comparisons for change in body weight (kg).

**Body weight (Kg) change from baseline to Week 12**						
**TZP 15 mg**	−0.62 (−2.83; 1.59)	−1.99 (−4.20; 0.22)	−2.14 (−5.19; 0.91)	−3.70 (−6.71; −0.69)	−5.59 (−8.86; −2.32)	−6.97 (−10.24; −3.70)
−0.61 (−2.81; 1.60)	**TZP 10 mg**	−1.38 (−3.58; 0.81)	.	−3.70 (−6.71; −0.69)	−4.25 (−7.47; −1.03)	−5.63 (−8.85; −2.41)
−1.99 (−4.20; 0.22)	−1.38 (−3.58; 0.81)	**TZP 5 mg**	.	−3.20 (−6.21; −0.19)	−1.87 (−5.09; 1.35)	−3.25 (−6.47; −0.03)
−2.14 (−5.19; 0.91)	−1.53 (−5.30; 2.23)	−0.15 (−3.92; 3.62)	**DULA 4.5 mg**	.	.	.
−4.40 (−7.17; −1.63)	−3.79 (−6.56; −1.03)	−2.41 (−5.17; 0.36)	−2.26 (−6.38; 1.86)	**DULA 0.75 mg**	.	.
−4.75 (−7.70; −1.80)	−4.15 (−7.08; −1.22)	−2.76 (−5.69; 0.17)	−2.61 (−6.86; 1.63)	−0.35 (−3.97; 3.26)	**DULA 1.5 mg**	−1.38 (−4.63; 1.87)
−6.13 (−9.08; −3.18)	−5.53 (−8.46; −2.60)	−4.14 (−7.07; −1.21)	−3.99 (−8.24; 0.25)	−1.73 (−5.35; 1.88)	−1.38 (−4.63; 1.87)	**TZP 1 mg**
**Body weight (Kg) change from baseline to Week 16**						
**TZP 15 mg**	−0.77 (−3.08; 1.54)	−2.68 (−4.98; −0.38)	−3.02 (−6.17; 0.13)	−6.41 (−9.82; −3.00)	−5.00 (−8.10; −1.90)	−8.05 (−11.52; −4.58)
−0.77 (−3.08; 1.54)	**TZP 10 mg**	−1.90 (−4.20; 0.40)	.	−4.55 (−7.96; −1.14)	−5.10 (−8.20; −2.00)	−6.19 (−9.66; −2.72)
−2.68 (−4.98; −0.38)	−1.91 (−4.21; 0.39)	**TZP 5 mg**	.	−1.91 (−5.29; 1.47)	−3.80 (−6.90; −0.70)	−3.55 (−6.99; −0.12)
−3.02 (−6.17; 0.13)	−2.25 (−6.15; 1.65)	−0.34 (−4.24; 3.56)	**DULA 4.5 mg**	.	.	.
−5.43 (−8.49; −2.36)	−4.66 (−7.72; −1.59)	−2.75 (−5.80; 0.30)	−2.41 (−6.80; 1.98)	**DULA 1.5 mg**	.	−1.64 (−5.05; 1.77)
−5.78 (−8.64; −2.92)	−5.01 (−7.87; −2.15)	−3.10 (−5.96; −0.25)	−2.76 (−7.01; 1.49)	−0.35 (−4.10; 3.39)	**DULA 0.75 mg**	.
−7.07 (−10.20; −3.94)	−6.30 (−9.43; −3.17)	−4.39 (−7.51; −1.28)	−4.05 (−8.49; 0.39)	−1.64 (−5.05; 1.77)	−1.29 (−5.08; 2.51)	**TZP 1 mg**

*Note:* The background colour and bold format highlights the study arms (TZP doses vs DULA doses) for better visualization and clarity.

Abbreviations: DULA, dulaglutide; kg, kilogramme; TZP, tirzepatide.

At Week 16, TZP 15 mg demonstrated more effectiveness in weight reduction compared to 5 mg TZP (MD: −2.68, 95% CI: [−4.98; −0.38], *p* = 0.0224). In contrast, DULA 0.75 mg and TZP 1 mg were associated with a reduced weight change from baseline (MD: 3.10, 95% CI: [0.25; 5.96], *p* = 0.0332; and MD: 4.39, 95% CI: [1.28; 7.51], *p* = 0.0057, respectively). However, the remaining comparisons showed no significant differences compared with 5 mg TZP. Based on SUCRA, TZP 15 mg was ranked as a potential treatment for weight reduction at Week 16 (SUCRA: 0.95). In contrast, DULA 0.75 mg and TZP 1 mg showed a relatively small effect on body weight (SUCRA: 0.22 and 0.08, respectively) (Figure 3C). The pooled studies showed substantial heterogeneity (*I* ^2^ = 85.4%). A network graph for the body weight (kg) change from baseline at Week 16 is illustrated in Figure 3D. All pairwise comparisons of relative TZP vs. DULA effects for mean change in HbA1C (%) at Weeks 12 and 24 are shown in Table 2.

**TABLE 2:** League table of network meta‐analysis displaying relative TZP vs. DULA effects for all pairwise comparisons for mean change in HbA1c (%).

**Mean change in HbA1c (%) from baseline to Week 12**						
**TZP 15 mg**	−0.08 (−0.21; 0.05)	−0.13 (−0.27; 0.01)	−0.26 (−0.41; −0.11)	−0.62 (−0.76; −0.48)	−1.22 (−3.20; 0.76)	−0.92 (−1.29; −0.55)
−0.08 (−0.21; 0.05)	**TZP 10 mg**	−0.06 (−0.20; 0.08)	.	−0.55 (−0.69; −0.41)	−1.10 (−3.07; 0.87)	−0.80 (−1.15; −0.45)
−0.13 (−0.27; 0.00)	−0.06 (−0.19; 0.08)	**TZP 5 mg**	.	−0.49 (−0.63; −0.35)	−0.80 (−3.57; 1.97)	−0.50 (−2.48; 1.48)
−0.26 (−0.41; −0.11)	−0.18 (−0.38; 0.02)	−0.13 (−0.33; 0.08)	**DULA 4.5 mg**	.	.	.
−0.62 (−0.76; −0.49)	−0.55 (−0.68; −0.41)	−0.49 (−0.63; −0.35)	−0.36 (−0.57; −0.16)	**DULA 0.75 mg**	.	.
−1.19 (−3.16; 0.78)	−1.12 (−3.09; 0.85)	−1.06 (−3.03; 0.91)	−0.93 (−2.91; 1.04)	−0.57 (−2.54; 1.40)	**TZP 1 mg**	0.30 (−1.68; 2.28)
−0.89 (−1.21; −0.57)	−0.82 (−1.13; −0.50)	−0.76 (−1.09; −0.43)	−0.63 (−0.99; −0.28)	−0.27 (−0.60; 0.06)	0.30 (−1.68; 2.28)	**DULA 1.5 mg**
**Mean change in HbA1c (%) from baseline to Week 24**						
**TZP 15 mg**	−0.18 (−0.39; 0.03)	−0.40 (−0.61; −0.19)	−0.70 (−0.93; −0.47)	−1.23 (−1.73; −0.73)	−1.14 (−1.38; −0.90)	−1.61 (−2.10; −1.13)
−0.18 (−0.39; 0.03)	**TZP 10 mg**	−0.23 (−0.44; −0.02)	.	−0.92 (−1.41; −0.44)	−0.99 (−1.23; −0.75)	−1.31 (−1.78; −0.84)
−0.40 (−0.62; −0.19)	−0.23 (−0.44; −0.02)	**TZP 5 mg**	.	−0.51 (−1.00; −0.02)	−0.81 (−1.05; −0.57)	−0.90 (−1.37; −0.43)
−0.70 (−0.93; −0.47)	−0.52 (−0.84; −0.21)	−0.30 (−0.61; 0.02)	**DULA 4.5 mg**	.	.	.
−1.08 (−1.50; −0.65)	−0.90 (−1.32; −0.48)	−0.67 (−1.09; −0.25)	−0.38 (−0.86; 0.11)	**DULA 1.5 mg**	.	−0.39 (−0.87; 0.10)
−1.17 (−1.40; −0.95)	−1.00 (−1.22; −0.77)	−0.77 (−1.00; −0.54)	−0.47 (−0.80; −0.15)	−0.10 (−0.55; 0.35)	**DULA 0.75 mg**	.
−1.46 (−1.87; −1.06)	−1.29 (−1.69; −0.88)	−1.06 (−1.46; −0.66)	−0.76 (−1.23; −0.29)	−0.39 (−0.87; 0.10)	−0.29 (−0.72; 0.14)	**TZP 1 mg**

Abbreviations: DULA, dulaglutide; TZP, tirzepatide.

#### 3.4.2 Mean Change in HbA1c (%) From Baseline

The network meta‐analysis evaluated the effect of different doses of TZP and DULA on the body mean change in HbA1c from baseline at Weeks 12 and 24. At Week 12, three studies were evaluated involving 1156 participants, and seven doses were assessed. At Week 24, three studies were evaluated involving 1128 participants, and seven doses were assessed.

**FIGURE 4:**
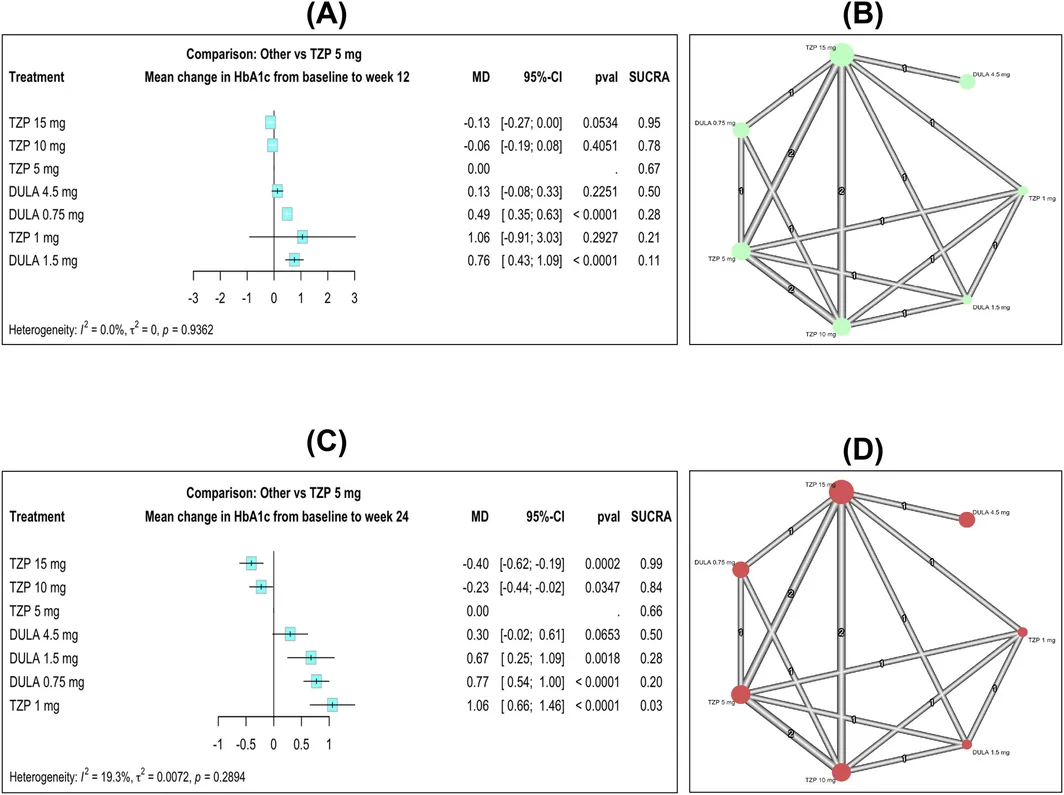
Glycemic‐related outcomes: (A) Forest plot for mean change in HbA1C (%) from baseline at week 12; (B) Net graph of network meta‐analysis mean change in HbA1C (%) from baseline at week 12; (C) Forest plot for mean change in HbA1C (%) from baseline at week 24; (D) Net graph of network meta‐analysis for mean change in HbA1C (%) from baseline at week 24.

At Week 12, DULA 0.75 mg and DULA 1.5 mg were associated with a potential increase in HbA1c compared to 5 mg TZP (MD: 0.49, 95% CI: [0.35; 0.63], *p* < 0.0001; and MD: 0.76, 95% CI: [0.43; 1.09], *p* < 0.0001, respectively). However, the remaining comparisons showed no significant differences compared with 5 mg TZP. Based on SUCRA, TZP 15 mg ranked as the most effective treatment (SUCRA: 0.95), and DULA 1.5 mg is among the smallest effects (SUCRA: 0.11) (Figure 4A). The pooled studies showed no heterogeneity was observed (*I* ^2^ = 0%). A network graph for the mean change in HbA1C (%) from baseline at week 12 is illustrated in Figure 4B.

At Week 24, TZP 15 mg demonstrated a potential reduction in HbA1c (MD: −0.40, 95% CI: [−0.62; −0.19], *p* = 0.0002), followed by TZP 10 mg (MD: −0.23, 95% CI: [−0.44; −0.02], *p* = 0.0347) compared with 5 mg TZP. In contrast, DULA 1.5 mg (MD: 0.67, 95% CI: [0.25; 1.09], *p* = 0.0018), DULA 0.75 mg (MD: 0.77, 95% CI: [0.54; 1.00], *p* < 0.0001), and TZP 1 mg (MD: 1.06, 95% CI: [0.66; 1.46], *p* < 0.0001) were associated with increased HbA1c compared to 5 mg TZP. Based on SUCRA, TZP 15 mg (SUCRA: 0.99) showed the greatest effect in reducing the HbA1c, followed by TZP 10 mg (SUCRA: 0.84) (Figure 4C). The pooled studies observed low heterogeneity (*I* ^2^ = 19.3%). A Network graph for the mean change in HbA1C (%) from baseline at week 24 is illustrated in Figure 4D.

#### 3.4.3 Adverse Events

**FIGURE 5:**
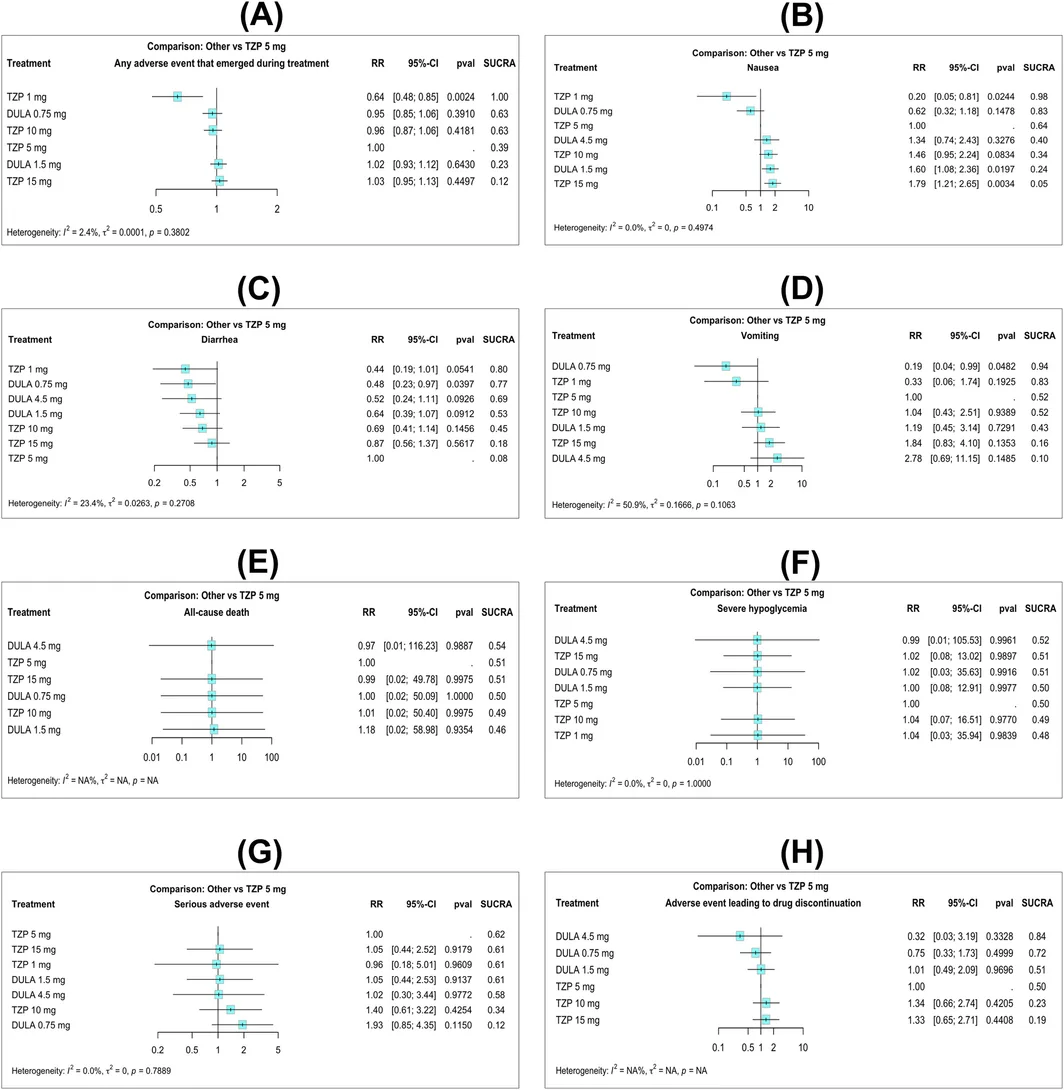
Safety outcomes: (A) Forest plot for any AE that emerged during treatment; (B) Forest plot for nausea; (C) Forest plot for diarrhoea; (D) Forest plot for vomiting; (E) Forest plot for all‐cause death; (F) Forest plot for severe hypoglycemia; (G) Forest plot for serious AE; (H) Forest plot for AE leading to drug discontinuation.

TZP 1 mg was associated with a lower risk of any AE that emerged during treatment compared to the 5 mg TZP (RR: 0.64, 95% CI: [0.48; 0.85], *p* = 0.0024; SUCRA: 1.00; *I* ^2^ = 2.4%) (Figure 5A), as well as nausea (RR: 0.20, 95% CI: [0.05; 0.81], *p* = 0.0244; SUCRA: 0.98; *I* ^2^ = 0%) (Figure 5B). No other comparisons reached statistical significance.

DULA 0.75 mg demonstrated a lower risk of diarrhoea compared with 5 mg TZP (RR: 0.48, 95% CI: [0.23; 0.97], *p* = 0.0397; *I* ^2^ = 23.4%) (Figure 5C), and was also associated with the lowest risk of vomiting (RR: 0.19, 95% CI: [0.04; 0.99], *p* = 0.0482; SUCRA: 0.94; *I* ^2^ = 50.9%) (Figure 5D). All remaining comparisons were not statistically significant. In contrast, DULA 1.5 mg (RR: 1.60, 95% CI: [1.08; 2.36], *p* = 0.0197; SUCRA: 0.24; *I* ^2^ = 0%) and TZP 15 mg (RR: 1.79, 95% CI: [1.21; 2.65], *p* = 0.0034; SUCRA: 0.05; *I* ^2^ = 0%) were associated with a higher risk of nausea compared to the 5 mg TZP (Figure 5B). No other significant differences were observed.

However, all comparisons showed no significant differences observed compared with 5 mg TZP in all‐cause death (Figure 5E), severe hypoglycemia (Figure 5F), serious AE (Figure 5G), headache (Figure S1), injection site reaction (Figure S2), dyspepsia (Figure S3), constipation (Figure S4), pancreatitis (Figure S5), AE leading to drug discontinuation (Figure 5H), and cardiovascular death, MI, or stroke (Figure S6). A Network graph for all safety outcomes is illustrated in Figure S7. Mortality and major adverse cardiovascular event estimates were based on limited data and wide confidence intervals and should therefore be interpreted with caution. All pairwise comparisons of relative TZP vs. DULA effects for safety outcomes are shown in Table S3.

### 3.5 Sensitivity Analysis

A sensitivity analysis was performed by excluding [11], which included a titrated TZP dose (up to 15 mg), patients with established ASCD, and also had the largest sample size compared to other studies. The sensitivity analysis was conducted for all‐cause death, severe hypoglycemia, serious AE, AE leading to drug discontinuation, any AE that emerged during treatment, pancreatitis, diarrhoea, nausea, and vomiting.

The results were consistent with the primary analysis for all‐cause death (Figure S8), severe hypoglycemia (Figure S9), serious AE (Figure S10), AE leading to drug discontinuation (Figure S11), any AE that emerged during treatment (Figure S12), pancreatitis (Figure S13), and vomiting (Figure S14). However, some discrepancies were observed. DULA 0.75 mg demonstrated no significant difference in the risk of diarrhoea compared with 5 mg TZP (Figure S15), in contrast to the primary analysis, which had shown a significant difference. Similarly, although TZP 1 mg was associated with a lower risk of nausea and TZP 15 mg with a higher risk of nausea (Figure S16) findings consistent with the primary analysis, DULA 1.5 mg showed no significant difference compared to 5 mg TZP in the sensitivity analysis, whereas the primary analysis had indicated a significant difference. Additionally, sensitivity analyses were conducted by excluding either Inagaki et al. [9] or Billings et al. [10]. Overall, the findings remained consistent with the primary analysis for the mean change in HbA1c from baseline to Week 24, all‐cause death, any adverse event that emerged during treatment, severe hypoglycemia, serious adverse event, headache, injection site reaction, dyspepsia, constipation, diarrhoea, pancreatitis, and adverse event leading to drug discontinuation.

For nausea, the results were consistent with the primary analysis across all comparisons after excluding either Inagaki et al. [9] or Billings et al. [10], except that the comparison involving DULA 1.5 mg was no longer statistically significant following the exclusion of Inagaki et al. [9], suggesting that the inclusion of that study influenced this outcome.

Regarding vomiting and the mean change in HbA1c from baseline to Week 12, the findings remained consistent with the primary analysis after excluding Billings et al. [10]. However, after excluding Inagaki et al. [9], none of the comparisons remained statistically significant, indicating that these results were sensitive to the inclusion of this study.

For body weight change from baseline at Weeks 12 and 16, the results remained consistent with the primary analysis after excluding Billings et al. [10]. In contrast, after excluding Inagaki et al. [9], all comparisons for body weight change at Week 12 became statistically significant, and the comparison involving DULA 1.5 mg also became statistically significant for body weight change at Week 16, indicating that these findings were sensitive to the inclusion of Inagaki et al. [9]. Sensitivity analyses excluding Billings et al. [10] (SURPASS‐SWITCH) or Inagaki et al. [9] (SURPASS J‐mono) are illustrated in Figures S17–S47.

### 3.6 Inconsistency

A design‐by‐treatment analysis did not reveal any significant global inconsistency among all outcomes (*p* > 0.05). However, a design‐by‐treatment analysis revealed significant global inconsistency in the following outcomes: headache and body weight change from baseline at Week 12 (*p* < 0.0001) and at Week 16 (*p* = 0.0011). Global inconsistency could not be assessed for the outcomes of all‐cause death, AE leading to drug discontinuation, and cardiovascular death, MI, stroke, or coronary revascularization. The node‐splitting method revealed no significant evidence of inconsistency for any of the outcomes (*p*‐value > 0.05).

## 4 Discussion

Our NMA compared multiple doses of TZP and DULA using evidence from four RCTs that enrolled 14,348 participants [8, 9, 10, 11]. The seven‐node network integrated direct and indirect evidence. This NMA allowed comparisons between doses that were not directly compared in individual trials. However, the findings should be interpreted with caution given the available evidence. Across outcomes, higher TZP doses showed higher effects for metabolic efficacy, whereas lower doses tended to show better gastrointestinal tolerability. TZP 15 mg was consistently ranked as effective potential option for early weight reduction, and TZP 15 mg ranked highest for HbA1c reduction at Week 12. In contrast, DULA 0.75 mg and TZP 1 mg frequently ranked better for tolerability outcomes. Sensitivity analyses indicated that the majority of results were not influenced by the exclusion of either the Billings et al. trial [10] or the Inagaki et al. trial [9]. However, the results for HbA1c at Week 12, body weight at Weeks 12 and 16, and nausea and vomiting were affected after excluding the Inagaki et al. trial [9], suggesting that this study had an important influence on these outcomes. SUCRA ranking may be helpful to summarize the relative likelihood of treatment efficacy, but does not claim superiority and should be read alongside the size and accuracy of the treatment effects and the consistency of the network. Accordingly, the SUCRA results should be considered exploratory rather than definitive findings of treatment superiority. Taken together, the findings support a clinically intuitive tradeoff: greater metabolic benefit with higher TZP exposure at the cost of more dose‐related gastrointestinal symptoms. This dose–response is biologically plausible. TZP activates both GIP and GLP‐1 receptors, improving glucose‐dependent insulin secretion, lowering glucagon, and promoting negative energy balance through appetite regulation and delayed gastric emptying [26, 27]. Higher doses increase receptor engagement and can therefore amplify both glycemic and weight effects, while also increasing the likelihood of gastrointestinal AE [28, 29].

For body weight, our network suggests clinically relevant separation between higher and lower TZP doses by Week 16. Even modest additional weight reduction can improve insulin sensitivity and may allow de‐escalation of therapies that promote weight gain. The substantial heterogeneity we observed for early weight outcomes is not unexpected. Weight trajectories in the first months are sensitive to titration schedules, baseline body mass index, diabetes duration, background therapy, and early gastrointestinal symptoms that may transiently alter intake. Trial evidence also supports that weight loss is dose‐dependent and potential effect with higher TZP doses compared with DULA [8, 9].

For HbA1c, the network produced more stable estimates with low heterogeneity. The superiority of TZP 15 mg and TZP 10 mg over TZP 5 mg at week 24 is clinically meaningful because differences of 0.2–0.4 percentage points can shift a number of patients across commonly used targets and reduce the need for early intensification. Dose ranging data show potential HbA1c reductions with increasing TZP dose versus DULA, supporting a graded efficacy signal [8]. In SURPASS‐SWITCH, switching from stable submaximal DULA to TZP resulted in greater HbA1c reduction than escalating DULA dosage by week 40, reinforcing the concept that moving to TZP and completing titration can yield additional glycemic benefit [10]. Broader head‐to‐head evidence is directionally consistent, as SURPASS‐2 demonstrated greater HbA1c reduction with TZP compared with semaglutide 1 mg, alongside a clear dose gradient across TZP doses [30].

However, an additional consideration when interpreting the efficacy results at Weeks 12 and 16 is that many participants, particularly those assigned to TZP 10 and 15 mg, were still undergoing the protocol‐mandated dose‐escalation phase and had not yet reached their maintenance dose. Consequently, the observed reductions in body weight and HbA1c at these early time points may underestimate the full therapeutic effects of the higher maintenance doses. These early findings should therefore be interpreted in the context of ongoing dose titration rather than steady‐state treatment.

The safety profile in our network was dominated by gastrointestinal AE. Lower doses were associated with fewer events in selected contrasts, including lower overall AE and nausea with TZP 1 mg and lower diarrhoea and vomiting with DULA 0.75 mg. Conversely, higher doses showed higher nausea risk, including TZP 15 mg and DULA 1.5 mg compared with TZP 5 mg. This pattern is consistent with slowed gastric emptying and central satiety signalling, both of which intensify with higher incretin exposure [31, 32]. These patterns support careful titration and early symptom management during escalation. Trials comparing TZP and DULA similarly identify gastrointestinal AE as the most common treatment‐emergent AE, while serious pancreatic events are uncommon in the reported follow‐up [10]. In dose‐escalation studies of DULA, higher doses improve HbA1c and weight with gastrointestinal AE remaining the dominant tolerability issue, which aligns with our network pattern for dose‐related nausea [33].

Although we did not detect differences in mortality or MACE within our network, this reflects limited power for rare outcomes in a network informed by four trials. External evidence helps contextualize these findings. DULA reduced MACE versus placebo in the REWIND trial [6]. Meta‐analyses of GLP‐1 RA cardiovascular outcomes trials have shown reductions in MACE and all‐cause mortality, with signals toward improved kidney outcomes [34, 35, 36, 37]. More recently, SURPASS‐CVOT compared TZP (dose‐adjusted up to 15 mg) with DULA 1.5 mg in patients with T2DM and established atherosclerotic cardiovascular disease [11]. The trial design and endpoints directly address the comparative cardiovascular safety question between these agents [11]. Inconsistency testing was reassuring. Global inconsistency was not detected for most outcomes, and local node splitting did not identify meaningful disagreement between direct and indirect evidence. The global inconsistency observed for early weight outcomes and headache reflects sparse connections and the variability of short‐term trajectories. However, rare outcomes, including all‐cause mortality, major cardiovascular events, severe hypoglycemia, serious adverse events, and pancreatitis, the available evidence remains limited and confidence intervals were wide. Therefore, the absence of statistically significant differences should not be interpreted as evidence of equivalent safety between TZP and DULA or between individual dose regimens.

Therapeutic options to address hyperglycemia and excess adiposity range from lifestyle and pharmacotherapy to metabolic and bariatric surgery [38]. Metabolic surgery offers the greatest durable weight loss and sustained glycemic remission in selected adults with T2DM [39, 40]. SMBS and IFSO recommend metabolic surgery for BMI > 35 kg/m^2^ and consider it for BMI 30–34.9 kg/m^2^ with metabolic disease, consistent with ADA guidance [40, 41]. Within pharmacotherapy, other GLP‐1 RAs, such as liraglutide and semaglutide, provide clinically meaningful HbA1c and weight reductions, and several agents have demonstrated cardiovascular benefit in outcome trials [42, 43, 44]. In addition, higher dose semaglutide 2.4 mg achieved clinically meaningful weight loss in adults with overweight or obesity and T2DM in STEP 2 trial, which helps benchmark the magnitude of weight effects seen with incretin‐based escalation strategies [45]. These broader data provide context for our dose level comparisons of TZP and DULA and support interpreting our findings as refinement within the injectable incretin class rather than a replacement for surgical strategies in eligible patients.

### 4.1 Clinical Implications

Guidelines recommend selecting glucose‐lowering therapy based on comorbidities and patient priorities rather than HbA1c alone. The ADA Standards of Care emphasize therapies that support weight loss and advise using GLP‐1 RA or a dual incretin agonist before basal insulin in many patients who need injectable therapy [46]. The ADA EASD consensus report stresses shared decision making and prioritizing agents that reduce cardiovascular and kidney risk [4]. For chronic kidney disease, KDIGO recommends a long‐acting GLP‐1 RA as part of risk‐based glycemic management in appropriate patients, particularly when additional glucose‐lowering is needed beyond foundational therapies [47]. Other guidance, including AACE and ESC focused recommendations, similarly prioritizes agents with weight and cardiovascular benefits in appropriate risk groups [48, 49].

In this context, our results provide dose‐specific comparisons that may improve treatment discussions. If the main goals are greater HbA1c reduction and weight loss, the repeated high ranking of TZP 15 and 10 mg supports dose‐escalation when patients can tolerate it, with an expected stepwise advantage over lower doses. These results may help clinicians communicate that treatment effects usually strengthen with higher doses and that early weight changes can be variable during dose‐escalation. When gastrointestinal tolerability is the main priority, initiating therapy at lower doses, such as DULA 0.75 mg or TZP 1 mg can be a reasonable approach, while acknowledging that glycemic and weight benefits may be more modest and later intensification may be required. Implementation in routine care is also influenced by local formulary availability and reimbursement decisions.

### 4.2 Strengths, Limitations, and Recommendations

This analysis has strengths that support internal validity. We included only RCTs (*n* = 4) and three of them were judged to have low risk of bias. The network approach enabled estimation across several TZP and DULA doses within a coherent framework, and random‐effects models accounted for between‐study variability. We examined transitivity, quantified heterogeneity, and evaluated inconsistency using both design by treatment interaction and node splitting methods, which strengthens inference for outcomes with stable networks. Moreover, we conducted sensitivity analysis by excluding the largest included trial enrolling patients with established cardiovascular disease [11], to assess the credibility and reliability of our findings; however, the results remained generally consistent with the primary analyses, with no changes in the overall interpretation of findings. These observations suggest that, although the precision of certain estimates may be influenced by the large trial, the principal conclusions of the network are not solely dependent on a single study. However, the findings should be interpreted with appropriate caution, particularly for outcomes supported by a limited number of studies. Moreover, to enhance the transitivity and transparency of our findings, multiple prespecified sensitivity analyses were performed, including exclusion of the SURPASS‐SWITCH and SURPASS J‐mono trials and restriction to treatment‐naïve populations, all of which supported the consistency of the primary results.

Limitations warrant caution. Only four RCTs informed the network, and several dose nodes were supported by a few comparisons. Key efficacy outcomes were assessed at early and intermediate time points, which limits inference about long‐term durability, late AE, and sustained treatment discontinuation. Weight outcomes showed substantial heterogeneity and global inconsistency at early time points, which reduces certainty about the exact size of short‐term differences. Trials also differed in titration schedules. However, we could not assess the escalating and switching doses of TZP and DULA, due to the limited number of included studies in efficacy and safety outcomes. Besides, there is no shared arm to conduct an analysis comparing these two approaches. In addition, the open‐label design of Billings et al. [10] may influence the reporting of subjective symptoms, such as nausea and headache, and switch trial designs may also be shaped by patient expectations and prior treatment experiences. Moreover, the observed heterogeneity is derived from differences in dosage approaches, which were not applicable to be assessed and upcoming RCTs to address this comprehensively. Some of the data were digitized from a graphical representation of the data using WebPlotDigitizer. There is a possibility of minor differences between the digitized and original values, although these are minimized by independent extraction and verification. Besides, the results are driven by one trial [11]; however, this was addressed by excluding this study through a sensitivity analysis to assess the reliability of our evidence. Specifically, this trial contributed data to all‐cause mortality, major adverse cardiovascular outcomes, overall adverse events, serious adverse events, treatment discontinuation due to adverse events, severe hypoglycemia, nausea, vomiting, diarrhoea, and pancreatitis. Consequently, estimates for these outcomes may have been influenced by the considerable statistical weight of this study. However, this trial did not contribute data to the majority of efficacy outcomes, including body‐weight change, HbA1c reduction, and cardiac events, which represent key measures of treatment effectiveness. When pooling the TZP arm from Nicholls et al. [11] (SURPASS‐CVOT) into this analysis, it should be noted that TZP was administered per a titrate‐to‐target strategy (up to 15 mg or maximum tolerated dose). In contrast, most other trials contributing TZP‐arm data to this pooled analysis may have used fixed‐dose regimens. This dosing heterogeneity should be considered when interpreting pooled effect estimates. Sensitivity analyses were conducted, but several safety and efficacy outcomes have wide 95% CI that should be interpreted cautiously. Although later time points, such as Week 40 and Week 52, may better reflect fully stabilized treatment effects, they could not be included because these outcomes were not consistently reported across all eligible trials, limiting the ability to perform a connected network meta‐analysis at later follow‐up.

Accordingly, future research should prioritize longer head‐to‐head trials comparing TZP with higher dose DULA that use harmonized titration schedules and consistent outcome definitions, and that also include patient‐reported outcomes and quality of life assessments. Individual patient data meta‐analysis could clarify effect modification by baseline body mass index, kidney function, and cardiovascular disease, and identify predictors of gastrointestinal intolerance and discontinuation. Comparative studies that integrate metabolic efficacy with cardiovascular, kidney, and safety endpoints will be particularly important to translate dose level differences into long‐term clinical decision making. We recommend further head‐to‐head trials to directly compare dose‐escalation and switching strategies between TZP and DULA. Additionally, a placebo‐anchored network would be a valuable direction for future research. Finally, the lack of statistically significant differences for rare safety outcomes, such as pancreatitis and mortality, does not mean the treatments are equally safe, as the low event rates limited the statistical power to detect potential differences between treatments.

## 5 Conclusion

Higher TZP doses, particularly 15 and 10 mg, were associated with greater HbA1c reduction and early weight loss, while lower doses of TZP and DULA appeared to have better gastrointestinal tolerability. Serious AE and mortality were not clearly different within the available network. Most findings were generally consistent in sensitivity analyses, with only HbA1c at Week 12, body weight at Weeks 12 and 16, and nausea and vomiting showing sensitivity to the inclusion of the Inagaki et al. [9] trial. The early efficacy findings at Weeks 12 and 16 should be interpreted with caution, as many participants were still undergoing dose escalation and had not yet reached their maintenance dose. These findings may suggest individualized titration that balances metabolic benefit with tolerability and patient priorities.

## Author Contributions

**Basel Abdelazeem:** supervision. **Ahmed W. Hageen:** conceptualization, investigation, writing – original draft, writing – review and editing, visualization, validation, methodology, software, formal analysis, project administration, data curation, supervision, resources. **Ahmed Hamdy Kandil:** data curation, resources. **Safir Eladawi:** data curation, resources. **Moatasem Abdelaziz:** data curation, resources. **Khalil Iyad:** data curation, resources. **Ahmed Farid Gadelmawla:** writing – original draft, writing – review and editing. **Mohamed Reyad Mohamed:** data curation, resources, software. **Karim Zinhom:** resources, data curation. **Hind Abdulhay:** data curation, resources. **Salma Bahnasy:** data curation, resources. **Ahmad Omar Saleh:** formal analysis, writing – original draft. **Mustafa Turkman:** project administration, writing – review and editing, writing – original draft. **Gregg C. Fonarow:** supervision.

## Funding

The authors have nothing to report.

## Ethics Statement

The authors have nothing to report.

## Consent

The authors have nothing to report.

## Conflicts of Interest

Dr. Fonarow has consulted for Abbott, Amgen, AstraZeneca, Bayer, Boehinger Ingelheim, Cytokinetics, Eli Lilly, Johnson & Johnson, Medtronic, Merck, Novartis, and Pfizer. The remaining authors declare no conflicts of interest.

## Supporting information


**Table S1:** Detailed search strategy for each database.
**Table S2:** Preferred Reporting Items for Systematic Reviews and Meta‐Analysis (PRISMA) checklist.
**Table S3:** League table of network meta‐analysis displaying relative TZP vs. DULA effects for all pairwise comparisons of all safety outcomes.
**Figure S1:** Forest plot for headache.
**Figure S2:** Forest plot for injection site reaction.
**Figure S3:** Forest plot for dyspepsia.
**Figure S4:** Forest plot for constipation.
**Figure S5:** Forest plot for pancreatitis.
**Figure S6:** Forest plot for cardiovascular death, MI, or stroke.
**Figure S7:** A Network graphs of for all safety outcomes: (A) any AE that emerged during treatment compared; (B) nausea; (C) diarrhoea; (D) vomiting; (E) all‐cause death; (F) severe hypoglycemia; (G) serious AE; (H) headache; (I) injection site reaction; (K) dyspepsia; (L) constipation; (M) AE leading to drug discontinuation; (N) cardiovascular death, MI, stroke or coronary revascularization.
**Figure S8:** Sensitivity analysis for all‐cause death by excluding Nicholas et al. 2025.
**Figure S9:** Sensitivity analysis for severe hypoglycemia by excluding Nicholas et al. 2025.
**Figure S10:** Sensitivity analysis for serious AE by excluding Nicholas et al. 2025.
**Figure S11:** Sensitivity analysis for AE leading to drug discontinuation by excluding Nicholas et al. 2025.
**Figure S12:** Sensitivity analysis for any AE that emerged during treatment by excluding Nicholas et al. 2025.
**Figure S13:** Sensitivity analysis for pancreatitis by excluding Nicholas et al. 2025.
**Figure S14:** Sensitivity analysis for vomiting by excluding Nicholas et al. 2025.
**Figure S15:** Sensitivity analysis for diarrhoea by excluding Nicholas et al. 2025.
**Figure S16:** Sensitivity analysis for nausea by excluding Nicholas et al. 2025.

**Table S3:** League table of network meta‐analysis displaying relative TZP vs. DULA effects for all pairwise comparisons of all safety outcomes.

**Figure S17:** Adverse event leading to drug discontinuation—sensitivity analysis excluding Billings et al. [10].
**Figure S18:** All‐cause death—sensitivity analysis excluding Billings et al. [10].
**Figure S19:** Body weight change from baseline at Week 12—sensitivity analysis excluding Billings et al. [10].
**Figure S20:** Body weight change from baseline at Week 16—sensitivity analysis excluding Billings et al. [10].
**Figure S21:** Constipation—sensitivity analysis excluding Billings et al. [10].
**Figure S22:** Diarrhoea—sensitivity analysis excluding Billings et al. [10].
**Figure S23:** Dyspepsia—sensitivity analysis excluding Billings et al. [10].
**Figure S24:** Headache—sensitivity analysis excluding Billings et al. [10].
**Figure S25:** Injection site reaction—sensitivity analysis excluding Billings et al. [10].
**Figure S26:** Mean change in HbA1c from baseline to Week 12—sensitivity analysis excluding Billings et al. [10].
**Figure S27:** Mean change in HbA1c from baseline to Week 24—sensitivity analysis excluding Billings et al. [10].
**Figure S28:** Nausea—sensitivity analysis excluding Billings et al. [10].
**Figure S29:** Pancreatitis—sensitivity analysis excluding Billings et al. [10].
**Figure S30:** Serious adverse event—sensitivity analysis excluding Billings et al. [10].
**Figure S31:** Severe hypoglycemia—sensitivity analysis excluding Billings et al. [10].
**Figure S32:** Vomiting—sensitivity analysis excluding Billings et al. [10].
**Figure S33:** Any treatment‐emergent adverse event—sensitivity analysis excluding Inagaki et al. [9].
**Figure S34:** Body weight change from baseline at Week 12—sensitivity analysis excluding Inagaki et al. [9].
**Figure S35:** Body weight change from baseline at Week 16—sensitivity analysis excluding Inagaki et al. [9].
**Figure S36:** Constipation—sensitivity analysis excluding Inagaki et al. [9].
**Figure S37:** Diarrhoea—sensitivity analysis excluding Inagaki et al. [9].
**Figure S38:** Dyspepsia—sensitivity analysis excluding Inagaki et al. [9].
**Figure S39:** Headache—sensitivity analysis excluding Inagaki et al. [9].
**Figure S40:** Injection site reaction—sensitivity analysis excluding Inagaki et al. [9].
**Figure S41:** Mean change in HbA1c from baseline to Week 12—sensitivity analysis excluding Inagaki et al. [9].
**Figure S42:** Mean change in HbA1c from baseline to Week 24—sensitivity analysis excluding Inagaki et al. [9].
**Figure S43:** Nausea—sensitivity analysis excluding Inagaki et al. [9].
**Figure S44:** Pancreatitis—sensitivity analysis excluding Inagaki et al. [9].
**Figure S45:** Serious adverse event—sensitivity analysis excluding Inagaki et al. [9].
**Figure S46:** Severe hypoglycemia—sensitivity analysis excluding Inagaki et al. [9].
**Figure S47:** Vomiting—sensitivity analysis excluding Inagaki et al. [9].

## Data Availability

The data that support the findings of this study are available from the corresponding author upon reasonable request.
