# Supplementary material for: Efficacy and Safety of Tirzepatide Versus Dulaglutide in Type 2 Diabetes With or Without Established Atherosclerotic Cardiovascular Disease: A Network Meta‐Analysis of Randomized Clinical Trials
Source: Endocrinol Diabetes Metab. 2026 Sep 7;9(5):e70313. doi: 10.1002/edm2.70313 (PMC13550879; doi:10.1002/edm2.70313)
Supplement: Supplementary file 1 [file EDM2-9-e70313-s001.docx]

**Supplementary File Part II**

**Title: Efficacy and Safety of Tirzepatide Versus Dulaglutide in Type 2 Diabetes with or without Established Atherosclerotic Cardiovascular Disease.: A Network Meta-Analysis of Randomized Clinical Trials**

**Table S3:** League table of network meta-analysis displaying relative TZP vs. DULA effects for all pairwise comparisons of all safety outcomes.

| **Cardiovascular death, MI, or stroke** | | | | | | |
| --- | --- | --- | --- | --- | --- | --- |
| **TZP 15 mg** | 0.99 (0.02 ; 49.78) | 0.99 (0.02 ; 49.78) | 0.89 (0.83 ; 0.96) | 0.33 (0.01 ; 8.02) | **.** | **.** |
| 0.99 (0.02 ; 49.78) | **DULA 0.75 mg** | 1.00 (0.02 ; 50.09) | **.** | 0.33 (0.01 ; 8.07) | **.** | **.** |
| 0.99 (0.02 ; 49.78) | 1.00 (0.02 ; 50.09) | **TZP 5 mg** | **.** | 0.33 (0.01 ; 8.07) | **.** | **.** |
| 0.89 (0.83 ; 0.96) | 0.90 (0.02 ; 45.09) | 0.90 (0.02 ; 45.09) | **DULA 1.5 mg** | . | **.** | **.** |
| 0.33 (0.01 ; 8.02) | 0.33 (0.01 ; 8.07) | 0.33 (0.01 ; 8.07) | 0.37 (0.02 ; 8.98) | **TZP 10 mg** | **.** | **.** |
| **All-cause death** | | | | | | |
| **TZP 15 mg** | 1.03 (0.06 ; 16.29) | 0.99 (0.02 ; 49.78) | 0.99 (0.02 ; 49.78) | 0.99 (0.02 ; 49.46) | 0.85 (0.76 ; 0.94) | **.** |
| 1.03 (0.06 ; 16.29) | **DULA 4.5 mg** | **.** | **.** | **.** | **.** | **.** |
| 0.99 (0.02 ; 49.78) | 0.97 (0.01 ; 116.23) | **TZP 5 mg** | 1.00 (0.02 ; 50.09) | 0.99 (0.02 ; 49.77) | **.** | **.** |
| 0.99 (0.02 ; 49.78) | 0.97 (0.01 ; 116.23) | 1.00 (0.02 ; 50.09) | **DULA 0.75 mg** | 0.99 (0.02 ; 49.77) | **.** | **.** |
| 0.99 (0.02 ; 49.46) | 0.96 (0.01 ; 115.50) | 0.99 (0.02 ; 49.77) | 0.99 (0.02 ; 49.77) | **TZP 10 mg** | **.** | **.** |
| 0.85 (0.76 ; 0.94) | 0.82 (0.05 ; 13.03) | 0.85 (0.02 ; 42.66) | 0.85 (0.02 ; 42.66) | 0.86 (0.02 ; 42.93) | **DULA 1.5 mg** | **.** |
| **AE that emerged during treatment** | | | | | | |
| **TZP 1 mg** | **.** | 0.64 (0.47 ; 0.87) | 0.69 (0.50 ; 0.94) | 0.68 (0.49 ; 0.93) | 0.59 (0.44 ; 0.79) | **.** |
| 0.67 (0.50 ; 0.90) | **DULA 0.75 mg** | 1.01 (0.89 ; 1.14) | 0.94 (0.84 ; 1.05) | **.** | 0.92 (0.83 ; 1.03) | **.** |
| 0.67 (0.50 ; 0.89) | 0.99 (0.88 ; 1.12) | **TZP 10 mg** | 0.96 (0.87 ; 1.06) | 1.06 (0.85 ; 1.31) | 0.92 (0.83 ; 1.01) | **.** |
| 0.64 (0.48 ; 0.85) | 0.95 (0.85 ; 1.06) | 0.96 (0.87 ; 1.06) | **TZP 5 mg** | 0.98 (0.78 ; 1.23) | 0.96 (0.87 ; 1.05) | **.** |
| 0.63 (0.47 ; 0.83) | 0.93 (0.84 ; 1.04) | 0.94 (0.85 ; 1.03) | 0.98 (0.89 ; 1.07) | **DULA 1.5 mg** | 0.99 (0.96 ; 1.01) | **.** |
| 0.62 (0.47 ; 0.82) | 0.92 (0.83 ; 1.02) | 0.93 (0.84 ; 1.02) | 0.97 (0.88 ; 1.06) | 0.99 (0.96 ; 1.01) | **TZP 15 mg** | **.** |
| **Severe hypoglycemia** | | | | | | |
| **DULA 1.5 mg** | **.** | 1.02 (0.02 ; 50.41) | **.** | 0.96 (0.02 ; 47.66) | 0.98 (0.66 ; 1.45) | 0.94 (0.02 ; 46.75) |
| 1.01 (0.02 ; 51.44) | **DULA 4.5 mg** | **.** | **.** | **.** | 0.97 (0.02 ; 48.65) | **.** |
| 1.00 (0.08 ; 12.91) | 0.99 (0.01 ; 105.53) | **TZP 5 mg** | 1.00 (0.02 ; 50.09) | 0.95 (0.02 ; 46.81) | 0.98 (0.06 ; 15.61) | 0.96 (0.06 ; 15.22) |
| 0.98 (0.03 ; 32.81) | 0.97 (0.01 ; 184.85) | 0.98 (0.03 ; 34.29) | **DULA 0.75 mg** | . | 1.01 (0.02 ; 50.40) | 0.99 (0.02 ; 49.77) |
| 0.96 (0.04 ; 25.35) | 0.95 (0.01 ; 156.08) | 0.96 (0.03 ; 33.40) | 0.98 (0.01 ; 84.67) | **TZP 1 mg** | 1.02 (0.02 ; 50.41) | 0.98 (0.02 ; 48.51) |
| 0.98 (0.66 ; 1.45) | 0.97 (0.02 ; 48.65) | 0.98 (0.08 ; 12.59) | 1.00 (0.03 ; 33.21) | 1.02 (0.04 ; 26.79) | **TZP 15 mg** | 0.97 (0.06 ; 15.45) |
| 0.96 (0.07 ; 12.39) | 0.95 (0.01 ; 101.31) | 0.96 (0.06 ; 15.22) | 0.98 (0.03 ; 34.21) | 1.00 (0.03 ; 34.49) | 0.98 (0.08 ; 12.50) | **TZP 10 mg** |
| **Serious AE** | | | | | | |
| **TZP 5 mg** | 1.01 (0.41 ; 2.52) | 0.47 (0.04 ; 5.06) | **.** | 0.33 (0.04 ; 3.05) | 0.70 (0.30 ; 1.61) | 0.57 (0.25 ; 1.32) |
| 0.95 (0.40 ; 2.30) | **TZP 15 mg** | 0.98 (0.14 ; 6.71) | 1.03 (0.44 ; 2.39) | 1.00 (0.95 ; 1.05) | 0.68 (0.30 ; 1.56) | 0.50 (0.21 ; 1.20) |

| 1.04 (0.20 ; 5.44) | 1.09 (0.24 ; 5.06) | **TZP 1 mg** | **.** | 0.69 (0.12 ; 3.98) | 0.65 (0.11 ; 3.75) | **.** |
| --- | --- | --- | --- | --- | --- | --- |
| 0.98 (0.29 ; 3.32) | 1.03 (0.44 ; 2.39) | 0.94 (0.16 ; 5.44) | **DULA 4.5 mg** | **.** | **.** | **.** |
| 0.95 (0.40 ; 2.29) | 1.00 (0.95 ; 1.05) | 0.91 (0.20 ; 4.24) | 0.97 (0.42 ; 2.26) | **DULA 1.5 mg** | 0.94 (0.20 ; 4.47) | **.** |
| 0.71 (0.31 ; 1.64) | 0.75 (0.34 ; 1.62) | 0.68 (0.14 ; 3.30) | 0.73 (0.23 ; 2.29) | 0.75 (0.34 ; 1.63) | **TZP 10 mg** | 0.72 (0.33 ; 1.57) |
| 0.52 (0.23 ; 1.17) | 0.54 (0.24 ; 1.23) | 0.50 (0.10 ; 2.57) | 0.53 (0.16 ; 1.71) | 0.55 (0.24 ; 1.23) | 0.73 (0.34 ; 1.54) | **DULA 0.75 mg** |
| **Headache** | | | | | | |
| **DULA 1.5 mg** | **.** | 0.48 (0.01 ; 15.57) | 0.94 (0.02 ; 39.84) | 0.20 (0.01 ; 5.36) | **.** | 0.51 (0.02 ; 16.48) |
| 0.57 (0.01 ; 36.50) | **DULA 4.5 mg** | **.** | **.** | 0.73 (0.05 ; 11.34) | **.** | **.** |
| 0.48 (0.01 ; 15.57) | 0.85 (0.02 ; 43.18) | **TZP 1 mg** | 1.96 (0.06 ; 63.39) | 0.41 (0.02 ; 8.20) | **.** | 1.06 (0.04 ; 25.64) |
| 0.42 (0.02 ; 11.05) | 0.73 (0.02 ; 24.54) | 0.86 (0.04 ; 16.88) | **TZP 10 mg** | 1.08 (0.12 ; 9.79) | 1.34 (0.09 ; 20.90) | 0.79 (0.09 ; 6.75) |
| 0.41 (0.02 ; 9.48) | 0.73 (0.05 ; 11.34) | 0.86 (0.05 ; 14.25) | 0.99 (0.11 ; 8.88) | **TZP 15 mg** | 0.33 (0.02 ; 6.63) | 0.79 (0.10 ; 6.55) |
| 0.36 (0.01 ; 15.23) | 0.64 (0.01 ; 29.17) | 0.75 (0.02 ; 24.23) | 0.87 (0.07 ; 11.45) | 0.87 (0.06 ; 12.52) | **DULA 0.75 mg** | 0.75 (0.05 ; 11.68) |
| 0.35 (0.01 ; 8.61) | 0.62 (0.02 ; 19.81) | 0.73 (0.04 ; 13.03) | 0.85 (0.10 ; 7.22) | 0.86 (0.10 ; 7.02) | 0.98 (0.08 ; 12.62) | **TZP 5 mg** |
| **Injection site reaction** | | | | | | |
| **DULA 4.5 mg** | **.** | **.** | 0.24 (0.03 ; 2.33) | **.** | **.** | **.** |
| 0.83 (0.03 ; 19.88) | **TZP 1 mg** | 0.35 (0.04 ; 3.55) | 1.02 (0.06 ; 16.92) | 0.25 (0.03 ; 2.30) | **.** | 0.17 (0.02 ; 1.51) |
| 0.39 (0.03 ; 4.55) | 0.46 (0.05 ; 4.12) | **TZP 5 mg** | 0.66 (0.24 ; 1.79) | 0.59 (0.24 ; 1.48) | 0.43 (0.14 ; 1.29) | 0.49 (0.11 ; 2.12) |
| 0.24 (0.03 ; 2.33) | 0.29 (0.03 ; 2.70) | 0.63 (0.24 ; 1.67) | **TZP 15 mg** | 0.91 (0.37 ; 2.22) | 0.92 (0.36 ; 2.36) | 0.17 (0.02 ; 1.48) |
| 0.23 (0.02 ; 2.62) | 0.28 (0.03 ; 2.39) | 0.60 (0.24 ; 1.50) | 0.95 (0.39 ; 2.31) | **TZP 10 mg** | 0.79 (0.30 ; 2.08) | 0.71 (0.18 ; 2.71) |
| 0.19 (0.02 ; 2.23) | 0.23 (0.02 ; 2.25) | 0.50 (0.18 ; 1.39) | 0.80 (0.32 ; 2.00) | 0.84 (0.33 ; 2.11) | **DULA 0.75 mg** | **.** |
| 0.14 (0.01 ; 1.98) | 0.17 (0.02 ; 1.51) | 0.37 (0.11 ; 1.30) | 0.59 (0.16 ; 2.24) | 0.62 (0.19 ; 2.06) | 0.74 (0.18 ; 2.98) | **DULA 1.5 mg** |
| **Dyspepsia** | | | | | | |
| **TZP 1 mg** | **.** | **.** | 0.21 (0.01 ; 5.31) | 0.35 (0.01 ; 10.51) | 0.20 (0.01 ; 5.21) | 0.08 (0.00 ; 1.66) |
| 0.67 (0.02 ; 21.28) | **DULA 0.75 mg** | **.** | **.** | 0.22 (0.03 ; 1.53) | 0.20 (0.03 ; 1.37) | 0.25 (0.04 ; 1.74) |
| 0.58 (0.02 ; 20.02) | 0.87 (0.07 ; 11.04) | **DULA 4.5 mg** | **.** | **.** | 0.27 (0.05 ; 1.50) | **.** |
| 0.21 (0.01 ; 5.31) | 0.31 (0.03 ; 3.48) | 0.36 (0.03 ; 4.46) | **DULA 1.5 mg** | 1.70 (0.17 ; 17.29) | 0.98 (0.12 ; 7.92) | 0.36 (0.06 ; 2.31) |
| 0.19 (0.01 ; 4.20) | 0.28 (0.04 ; 1.81) | 0.32 (0.04 ; 2.70) | 0.89 (0.13 ; 5.94) | **TZP 5 mg** | 0.80 (0.23 ; 2.77) | 0.64 (0.19 ; 2.18) |
| 0.15 (0.01 ; 3.37) | 0.23 (0.04 ; 1.48) | 0.27 (0.05 ; 1.50) | 0.74 (0.12 ; 4.66) | 0.83 (0.24 ; 2.89) | **TZP 15 mg** | 0.77 (0.24 ; 2.45) |
| 0.11 (0.01 ; 2.35) | 0.17 (0.03 ; 1.09) | 0.19 (0.02 ; 1.56) | 0.54 (0.09 ; 3.13) | 0.61 (0.18 ; 2.06) | 0.73 (0.23 ; 2.34) | **TZP 10 mg** |
| **Constipation** | | | | | | |
| **TZP 1 mg** | **.** | **.** | 0.35 (0.04 ; 3.26) | 0.51 (0.05 ; 5.52) | 0.53 (0.05 ; 5.73) | 0.16 (0.02 ; 1.33) |
| 0.40 (0.05 ; 3.33) | **DULA 0.75 mg** | **.** | **.** | 0.78 (0.41 ; 1.48) | 0.71 (0.38 ; 1.33) | 0.60 (0.33 ; 1.11) |
| 0.37 (0.04 ; 3.42) | 0.91 (0.32 ; 2.56) | **DULA 4.5 mg** | **.** | 0.89 (0.39 ; 2.02) | **.** | **.** |

| 0.35 (0.04 ; 3.26) | 0.86 (0.22 ; 3.41) | 0.94 (0.20 ; 4.49) | **DULA 1.5 mg** | 1.47 (0.25 ; 8.60) | 1.53 (0.26 ; 8.93) | 0.47 (0.12 ; 1.83) |
| --- | --- | --- | --- | --- | --- | --- |
| 0.33 (0.04 ; 2.61) | 0.81 (0.43 ; 1.53) | 0.89 (0.39 ; 2.02) | 0.94 (0.25 ; 3.56) | **TZP 15 mg** | 0.92 (0.53 ; 1.62) | 0.70 (0.41 ; 1.19) |
| 0.30 (0.04 ; 2.39) | 0.74 (0.40 ; 1.39) | 0.82 (0.30 ; 2.21) | 0.87 (0.23 ; 3.25) | 0.92 (0.52 ; 1.61) | **TZP 5 mg** | 0.76 (0.45 ; 1.28) |
| 0.23 (0.03 ; 1.74) | 0.56 (0.31 ; 1.02) | 0.61 (0.23 ; 1.63) | 0.65 (0.18 ; 2.33) | 0.69 (0.40 ; 1.17) | 0.75 (0.45 ; 1.26) | **TZP 10 mg** |
| **Nausea** | | | | | | |
| **TZP 1 mg** | **.** | 0.19 (0.04 ; 0.83) | **.** | 0.18 (0.04 ; 0.76) | 0.13 (0.03 ; 0.54) | 0.10 (0.02 ; 0.39) |
| 0.32 (0.07 ; 1.42) | **DULA 0.75 mg** | 0.63 (0.32 ; 1.26) | **.** | 0.38 (0.21 ; 0.72) | **.** | 0.38 (0.20 ; 0.71) |
| 0.20 (0.05 ; 0.81) | 0.62 (0.32 ; 1.18) | **TZP 5 mg** | **.** | 0.70 (0.46 ; 1.08) | 0.68 (0.35 ; 1.32) | 0.56 (0.37 ; 0.83) |
| 0.15 (0.03 ; 0.63) | 0.46 (0.22 ; 0.98) | 0.74 (0.41 ; 1.34) | **DULA 4.5 mg** | **.** | **.** | 0.75 (0.48 ; 1.17) |
| 0.14 (0.03 ; 0.55) | 0.42 (0.23 ; 0.78) | 0.69 (0.45 ; 1.05) | 0.92 (0.52 ; 1.62) | **TZP 10 mg** | 0.73 (0.37 ; 1.42) | 0.80 (0.56 ; 1.15) |
| 0.12 (0.03 ; 0.49) | 0.39 (0.21 ; 0.71) | 0.63 (0.42 ; 0.93) | 0.84 (0.54 ; 1.32) | 0.91 (0.64 ; 1.30) | **DULA 1.5 mg** | 0.89 (0.84 ; 0.95) |
| 0.11 (0.03 ; 0.44) | 0.35 (0.19 ; 0.63) | 0.56 (0.38 ; 0.82) | 0.75 (0.48 ; 1.17) | 0.81 (0.57 ; 1.16) | 0.89 (0.84 ; 0.95) | **TZP 15 mg** |
| **Vomiting** | | | | | | |
| **DULA 0.75 mg** | **.** | 0.15 (0.03 ; 0.82) | 0.25 (0.04 ; 1.40) | **.** | 0.11 (0.02 ; 0.55) | **.** |
| 0.58 (0.07 ; 5.23) | **TZP 1 mg** | 0.53 (0.08 ; 3.32) | 0.25 (0.04 ; 1.34) | 0.42 (0.07 ; 2.47) | 0.15 (0.03 ; 0.75) | **.** |
| 0.19 (0.04 ; 0.99) | 0.33 (0.06 ; 1.74) | **TZP 5 mg** | 0.96 (0.39 ; 2.36) | 0.79 (0.18 ; 3.49) | 0.48 (0.21 ; 1.10) | **.** |
| 0.19 (0.04 ; 0.97) | 0.32 (0.06 ; 1.63) | 0.97 (0.40 ; 2.35) | **TZP 10 mg** | 1.69 (0.45 ; 6.34) | 0.50 (0.23 ; 1.12) | **.** |
| 0.16 (0.03 ; 0.90) | 0.28 (0.05 ; 1.43) | 0.84 (0.32 ; 2.23) | 0.87 (0.34 ; 2.22) | **DULA 1.5 mg** | 0.64 (0.33 ; 1.27) | **.** |
| 0.11 (0.02 ; 0.52) | 0.18 (0.04 ; 0.87) | 0.54 (0.24 ; 1.21) | 0.56 (0.26 ; 1.22) | 0.65 (0.33 ; 1.26) | **TZP 15 mg** | 0.66 (0.21 ; 2.06) |
| 0.07 (0.01 ; 0.49) | 0.12 (0.02 ; 0.83) | 0.36 (0.09 ; 1.44) | 0.37 (0.09 ; 1.47) | 0.43 (0.11 ; 1.59) | 0.66 (0.21 ; 2.06) | **DULA 4.5 mg** |
| **Diarrhea** | | | | | | |
| **TZP 1 mg** | **.** | **.** | 0.81 (0.31 ; 2.12) | 0.57 (0.23 ; 1.42) | 0.42 (0.18 ; 0.99) | 0.57 (0.23 ; 1.39) |
| 0.93 (0.33 ; 2.60) | **DULA 0.75 mg** | **.** | **.** | 0.78 (0.34 ; 1.78) | 0.61 (0.28 ; 1.35) | 0.41 (0.19 ; 0.85) |
| 0.85 (0.31 ; 2.32) | 0.91 (0.35 ; 2.35) | **DULA 4.5 mg** | **.** | **.** | 0.60 (0.32 ; 1.10) | **.** |
| 0.69 (0.30 ; 1.57) | 0.74 (0.34 ; 1.59) | 0.81 (0.41 ; 1.60) | **DULA 1.5 mg** | 0.71 (0.31 ; 1.64) | 0.72 (0.54 ; 0.98) | 0.71 (0.31 ; 1.61) |
| 0.64 (0.28 ; 1.50) | 0.69 (0.33 ; 1.47) | 0.76 (0.35 ; 1.66) | 0.94 (0.54 ; 1.61) | **TZP 10 mg** | 0.76 (0.46 ; 1.26) | 0.70 (0.42 ; 1.16) |
| 0.51 (0.23 ; 1.12) | 0.54 (0.26 ; 1.12) | 0.60 (0.32 ; 1.10) | 0.74 (0.55 ; 0.99) | 0.79 (0.48 ; 1.28) | **TZP 15 mg** | 0.92 (0.58 ; 1.47) |
| 0.44 (0.19 ; 1.01) | 0.48 (0.23 ; 0.97) | 0.52 (0.24 ; 1.11) | 0.64 (0.39 ; 1.07) | 0.69 (0.41 ; 1.14) | 0.87 (0.56 ; 1.37) | **TZP 5 mg** |
| **Pancreatitis** | | | | | | |
| **TZP 10 mg** | 1.02 (0.02 ; 50.41) | 1.01 (0.02 ; 50.40) | 1.06 (0.02 ; 52.35) | **.** | 0.53 (0.04 ; 6.27) | 0.38 (0.04 ; 4.16) |
| 1.01 (0.03 ; 33.17) | **TZP 1 mg** | **.** | 1.04 (0.02 ; 51.36) | **.** | 1.02 (0.02 ; 50.41) | 0.21 (0.01 ; 4.30) |
| 1.01 (0.03 ; 33.49) | 1.00 (0.01 ; 72.03) | **DULA 0.75 mg** | **.** | **.** | 0.34 (0.01 ; 8.17) | 1.00 (0.02 ; 50.09) |
| 0.56 (0.05 ; 5.83) | 0.55 (0.02 ; 13.70) | 0.55 (0.02 ; 12.69) | **DULA 1.5 mg** | **.** | 0.95 (0.62 ; 1.47) | 0.20 (0.01 ; 4.15) |

| 0.54 (0.01 ; 51.04) | 0.53 (0.00 ; 83.60) | 0.53 (0.00 ; 78.89) | 0.96 (0.02 ; 49.37) | **DULA 4.5 mg** | 0.97 (0.02 ; 48.65) | **.** |
| --- | --- | --- | --- | --- | --- | --- |
| 0.52 (0.05 ; 5.34) | 0.52 (0.02 ; 12.71) | 0.52 (0.02 ; 11.60) | 0.94 (0.61 ; 1.44) | 0.97 (0.02 ; 48.65) | **TZP 15 mg** | 0.73 (0.08 ; 6.51) |
| 0.27 (0.03 ; 2.80) | 0.27 (0.01 ; 5.38) | 0.27 (0.01 ; 7.49) | 0.49 (0.08 ; 3.12) | 0.51 (0.01 ; 38.27) | 0.52 (0.08 ; 3.27) | **TZP 5 mg** |
| **AE leading to drug discontinuation** | | | | | | |
| **DULA 4.5 mg** | **.** | **.** | **.** | **.** | 0.24 (0.03 ; 2.15) | **.** |
| 0.43 (0.04 ; 4.35) | **DULA 0.75 mg** | **.** | 0.75 (0.33 ; 1.73) | 0.56 (0.25 ; 1.23) | 0.57 (0.26 ; 1.24) | **.** |
| 0.32 (0.04 ; 2.81) | 0.74 (0.33 ; 1.63) | **DULA 1.5 mg** | **.** | **.** | 0.77 (0.70 ; 0.84) | **.** |
| 0.32 (0.03 ; 3.19) | 0.75 (0.33 ; 1.73) | 1.01 (0.49 ; 2.09) | **TZP 5 mg** | 0.75 (0.36 ; 1.52) | 0.75 (0.37 ; 1.54) | **.** |
| 0.24 (0.02 ; 2.34) | 0.56 (0.25 ; 1.23) | 0.76 (0.39 ; 1.47) | 0.75 (0.36 ; 1.52) | **TZP 10 mg** | 1.01 (0.52 ; 1.95) | **.** |
| 0.24 (0.03 ; 2.15) | 0.57 (0.26 ; 1.24) | 0.77 (0.70 ; 0.84) | 0.75 (0.37 ; 1.54) | 1.01 (0.52 ; 1.95) | **TZP 15 mg** | **.** |

**AE;** adverse events, **MI;** myocardial infarction, **TZP;** tirzepatide, **DULA;** dulaglutide.
