# Supplementary material for: Efficacy and Safety of Tirzepatide Versus Dulaglutide in Type 2 Diabetes With or Without Established Atherosclerotic Cardiovascular Disease: A Network Meta‐Analysis of Randomized Clinical Trials
Source: Endocrinol Diabetes Metab. 2026 Sep 7;9(5):e70313. doi: 10.1002/edm2.70313 (PMC13550879; doi:10.1002/edm2.70313)
Supplement: Supplementary file 2 [file EDM2-9-e70313-s002.docx]

Supplementary Part I Appendix

Efficacy and Safety of Tirzepatide Versus Dulaglutide in Type 2 Diabetes with or without Established Atherosclerotic Cardiovascular Disease: A Network Meta-Analysis of Randomized Clinical Trials.

**Authors:**
Ahmed W. Hageen, MBBCh^#1^; Ahmed Farid Gadelmawla, MD^#2,3^; Ahmad Omar Saleh, MD^4^; Salma Bahnasy, MBBCh^5^; Safir Eladawi, MBBCh^6^; Moatasem Abdelaziz, MBBCh^7^; Khalil Iyad, MBBCh^8^; Ahmed Hamdy Kandil, MD^9^; Karim Zinhom, MBBCh^10^; Mohamed Reyad Mohamed, MD^11^; Hind Abdulhay, MD^12^; Mustafa Turkman, MD^13,14^; Basel Abdelazeem, MD^15^; Gregg C. Fonarow, MD, FACC^16^.

**Affiliations:**

1. Faculty of Medicine, Tanta University, Tanta, Egypt.
2. Faculty of Medicine, Menoufia University, Menoufia, Egypt.
3. Medical Research Group of Egypt (MRGE), Negida Academy, Arlington, MA, USA.
4. Faculty of medicine, The university of Jordan, Amman, Jordan.
5. Matruh Specialized Center for Cardiac Surgery and Interventional Catheter, Marsa Matruh, Egypt.
6. Faculty of Medicine, Ain Shams University, Cairo, Egypt.
7. Faculty of Medicine, Kafr Elsheikh University, Kafr Elsheikh, Egypt.
8. Faculty of Medicine, Alexandria University, Alexandria, Egypt.
9. Faculty of Medicine, Benha University, Benha, Egypt.
10. Faculty of Medicine, October 6 University, Giza, Egypt.
11. Internal Medicine Department, University of Arizona College of Medicine–Phoenix, Phoenix, Arizona, USA.
12. Faculty of Medicine, Mansoura University, Dakahlia, Egypt.
13. Faculty of Medicine, Michigan State University, East Lansing, Michigan, USA.
14. Division of Pulmonary and Critical Care, University of Toledo, Toledo, Ohio, USA.
15. Department of Cardiology, West Virginia University, Morgantown, WV, USA.
16. Division of Cardiology, University of California Los Angeles, Los Angeles, California, USA.

**#Both authors contributed equally and shared co-first authorship position.**

| **APPENDIX Page** | |
| --- | --- |
| **TABLES** | |
| **Table S1.** Detailed search strategy for each database. | 5 |
| **Table S2.** Preferred Reporting Items for Systematic Reviews and Meta-Analysis (PRISMA) checklist. | 6 |
| **Table S3.** League table of network meta-analysis displaying relative TZP vs. DULA effects for all pairwise comparisons of all safety outcomes. | 8 |
| **FIGURES** | |
| **Figure S1.** Forest plot for headache. | 8 |
| **Figure S2.** Forest plot for injection site reaction. | 8 |
| **Figure S3.** Forest plot for dyspepsia. | 9 |
| **Figure S4.** Forest plot for constipation | 9 |
| **Figure S5**. Forest plot for pancreatitis. | 10 |
| **Figure S6**. Forest plot for cardiovascular death, MI, or stroke. | 10 |
| **Figure S7**. A Network graph of for all safety outcomes. | 11 |
| **Figure S8.** Sensitivity analysis for all-cause death by excluding Nicholls et al. 2025 | 12 |
| **Figure S9.** Sensitivity analysis for severe hypoglycemia by excluding Nicholls et al. 2025 | 12 |
| **Figure S10.** Sensitivity analysis for serious AE by excluding Nicholls et al. 2025 | 12 |
| **Figure S11.** Sensitivity analysis for AE leading to drug discontinuation by excluding Nicholls et al. 2025 | 13 |
| **Figure S12.** Sensitivity analysis for any AE that emerged during treatment by excluding Nicholls et al. 2025 | 13 |
| **Figure S13.** Sensitivity analysis for pancreatitis by excluding Nicholls et al. 2025 | 13 |
| **Figure S14.** Sensitivity analysis for vomiting by excluding Nicholls et al. 2025 | 14 |
| **Figure S15.** Sensitivity analysis for diarrhea by excluding Nicholls et al. 2025 | 14 |
| **Figure S16.** Sensitivity analysis for nausea by excluding Nicholls et al. 2025 | 14 |
| **Figures S17-S47.** Sensitivity Analyses Excluding Billings et al. 2025 or Inagaki et al. 2022 | 15 |

***Abbreviations.***

**TZP;** tirzepatide, **DULA;** dulaglutide, **AE;** adverse events.

**Table S1:** Detailed search strategy for each database.

| **Database** | **Search Terms** | **Search Field** | | **Results** |
| --- | --- | --- | --- | --- |
| **PubMed** | (((Type 2 diabetes mellitus) OR (Type II diabetes mellitus) OR (T2DM) OR (T2D) OR (Adult-onset diabetes) OR (Non-insulin-dependent diabetes mellitus) OR (NIDDM) OR (Diabetes mellitus type 2) OR (Diabetes type 2) OR (Diabetes Mellitus, Type 2)) AND ((Tirzepatide) OR (LY3298176) OR (LY-3298176) OR (Mounjaro) OR (Zepbound))) AND ((Dulaglutide) OR (LY2189265) OR (LY-2189265)) | All Fields | 97 | |
| **WOS** | (((Type 2 diabetes mellitus) OR (Type II diabetes mellitus) OR (T2DM) OR (T2D) OR (Adult-onset diabetes) OR (Non-insulin-dependent diabetes mellitus) OR (NIDDM) OR (Diabetes mellitus type 2) OR (Diabetes type 2) OR (Diabetes Mellitus, Type 2)) AND ((Tirzepatide) OR (LY3298176) OR (LY-3298176) OR (Mounjaro) OR (Zepbound))) AND ((Dulaglutide) OR (LY2189265) OR (LY-2189265)) | All Fields | 124 | |
| **Scopus** | (((Type 2 diabetes mellitus) OR (Type II diabetes mellitus) OR (T2DM) OR (T2D) OR (Adult-onset diabetes) OR (Non-insulin-dependent diabetes mellitus) OR (NIDDM) OR (Diabetes mellitus type 2) OR (Diabetes type 2) OR (Diabetes Mellitus, Type 2)) AND ((Tirzepatide) OR (LY3298176) OR (LY-3298176) OR (Mounjaro) OR (Zepbound))) AND ((Dulaglutide) OR (LY2189265) OR (LY-2189265)) | Title, Abstract, Keywords | 463 | |
| **Cochrane** | (((Type 2 diabetes mellitus) OR (Type II diabetes mellitus) OR (T2DM) OR (T2D) OR (Adult-onset diabetes) OR (Non-insulin-dependent diabetes mellitus) OR (NIDDM) OR (Diabetes mellitus type 2) OR (Diabetes type 2) OR (Diabetes Mellitus, Type 2)) AND ((Tirzepatide) OR (LY3298176) OR (LY-3298176) OR (Mounjaro) OR (Zepbound))) AND ((Dulaglutide) OR (LY2189265) OR (LY-2189265)) | All Fields | 42 | |

**Table S2.** Preferred Reporting Items for Systematic Reviews and Meta-Analysis (PRISMA) checklist.

| **Section and Topic** | **Item #** | **Checklist item** | **Page where item is reported** |
| --- | --- | --- | --- |
| **TITLE** | | |  |
| Title | 1 | Identify the report as a systematic review. | 1 |
| **ABSTRACT** | | |  |
| Abstract | 2 | See the PRISMA 2020 for Abstracts checklist. | 4 |
| **INTRODUCTION** | | |  |
| Rationale | 3 | Describe the rationale for the review in the context of existing knowledge. | 6 |
| Objectives | 4 | Provide an explicit statement of the objective(s) or question(s) the review addresses. | 7 |
| **METHODS** | | |  |
| Eligibility criteria | 5 | Specify the inclusion and exclusion criteria for the review and how studies were grouped for the syntheses. | 8 |
| Information sources | 6 | Specify all databases, registers, websites, organisations, reference lists and other sources searched or consulted to identify studies. Specify the date when each source was last searched or consulted. | 7&8 |
| Search strategy | 7 | Present the full search strategies for all databases, registers and websites, including any filters and limits used. | 7 |
| Selection process | 8 | Specify the methods used to decide whether a study met the inclusion criteria of the review, including how many reviewers screened each record and each report retrieved, whether they worked independently, and if applicable, details of automation tools used in the process. | 8 |
| Data collection process | 9 | Specify the methods used to collect data from reports, including how many reviewers collected data from each report, whether they worked independently, any processes for obtaining or confirming data from study investigators, and if applicable, details of automation tools used in the process. | 7&8 |
| Data items | 10a | List and define all outcomes for which data were sought. Specify whether all results that were  compatible with each outcome domain in each study were sought (e.g. for all measures, time points, analyses), and if not, the methods used to decide which results to collect. | 8 |

| **Section and Topic** | **Item #** | **Checklist item** | **Page where item is**  **reported** |
| --- | --- | --- | --- |
|  | 10b | List and define all other variables for which data were sought (e.g. participant and intervention characteristics, funding sources). Describe any assumptions made about any missing or unclear information. | 8 |
| Study risk of bias assessment | 11 | Specify the methods used to assess risk of bias in the included studies, including details of the tool(s) used, how many reviewers assessed each study and whether they worked independently, and if applicable, details of automation tools used in the process. | 10 |
| Effect  measures | 12 | Specify for each outcome the effect measure(s) (e.g. risk ratio, mean difference) used in the synthesis or presentation of results. | 10 |
| Synthesis methods | 13a | Describe the processes used to decide which studies were eligible for each synthesis (e.g. tabulating the study intervention characteristics and comparing against the planned groups for each synthesis (item #5)). | 10 |
|  | 13b | Describe any methods required to prepare the data for presentation or synthesis, such as handling of missing summary statistics, or data conversions. | 10 |
|  | 13c | Describe any methods used to tabulate or visually display results of individual studies and syntheses. | 10 |
|  | 13d | Describe any methods used to synthesize results and provide a rationale for the choice(s). If  meta-analysis was performed, describe the model(s), method(s) to identify the presence and extent of statistical heterogeneity, and software package(s) used. | 10 |
|  | 13e | Describe any methods used to explore possible causes of heterogeneity among study results (e.g. subgroup analysis, meta-regression). | 10 |
|  | 13f | Describe any sensitivity analyses conducted to assess robustness of the synthesized results. | NA |
| Reporting bias assessment | 14 | Describe any methods used to assess risk of bias due to missing results in a synthesis (arising from reporting biases). | 10 &13 |
| Certainty assessment | 15 | Describe any methods used to assess certainty (or confidence) in the body of evidence for an outcome. | NA |
| **RESULTS** | | |  |

| **Section and Topic** | **Item #** | **Checklist item** | **Page where item is**  **reported** |
| --- | --- | --- | --- |
| Study selection | 16a | Describe the results of the search and selection process, from the number of records identified in the search to the number of studies included in the review, ideally using a flow diagram. | 8&12 |
|  | 16b | Cite studies that might appear to meet the inclusion criteria, but which were excluded, and explain why they were excluded. | 13 |
| Study  characteristics | 17 | Cite each included study and present its characteristics. | 13 |
| Risk of bias in studies | 18 | Present assessments of risk of bias for each included study. | 13 |
| Results of individual studies | 19 | For all outcomes, present, for each study: (a) summary statistics for each group (where appropriate) and (b) an effect estimate and its precision (e.g. confidence/credible interval), ideally using structured tables or plots. | 10-12 |
| Results of syntheses | 20a | For each synthesis, briefly summarise the characteristics and risk of bias among contributing studies. | 13 |
|  | 20b | Present results of all statistical syntheses conducted. If meta-analysis was done, present for each the summary estimate and its precision (e.g. confidence/credible interval) and measures of statistical heterogeneity. If comparing groups, describe the direction of the effect. | 12-18 |
|  | 20c | Present results of all investigations of possible causes of heterogeneity among study results. | 18 |
|  | 20d | Present results of all sensitivity analyses conducted to assess the robustness of the synthesized results. | NA |
| Reporting biases | 21 | Present assessments of risk of bias due to missing results (arising from reporting biases) for each synthesis assessed. | 13 |
| Certainty of evidence | 22 | Present assessments of certainty (or confidence) in the body of evidence for each outcome assessed. | NA |
| **DISCUSSION** | | |  |
| Discussion | 23a | Provide a general interpretation of the results in the context of other evidence. | 19 |
|  | 23b | Discuss any limitations of the evidence included in the review. | 24 |

| **Section and Topic** | **Item #** | **Checklist item** | **Page where item is**  **reported** |
| --- | --- | --- | --- |
|  | 23c | Discuss any limitations of the review processes used. | 24 |
|  | 23d | Discuss implications of the results for practice, policy, and future research. | 22 & 23 |
| **OTHER INFORMATION** | | |  |
| Registration and protocol | 24a | Provide registration information for the review, including register name and registration number, or state that the review was not registered. | 7 |
|  | 24b | Indicate where the review protocol can be accessed, or state that a protocol was not prepared. | 7 |
|  | 24c | Describe and explain any amendments to information provided at registration or in the protocol. | 7 |
| Support | 25 | Describe sources of financial or non-financial support for the review, and the role of the funders or sponsors in the review. | 26 |
| Competing interests | 26 | Declare any competing interests of review authors. | 26 |
| Availability of data, code and other materials | 27 | Report which of the following are publicly available and where they can be found: template data collection forms; data extracted from included studies; data used for all analyses; analytic code; any other materials used in the review. | 27 |

**Reference:**	Page MJ, McKenzie JE, Bossuyt PM, Boutron I, Hoffmann TC, Mulrow CD, et al. The PRISMA 2020 statement: an updated guideline for reporting systematic reviews. BMJ 2021;372:n71. doi: 10.1136/bmj.n

**Table S3.** League table of network meta-analysis displaying relative TZP vs. DULA effects for all pairwise comparisons of all safety outcomes.

**Note:** **Table S3 is attached in Supplementary File Part II.**

**Figure S1.** Forest plot for headache.

**Figure S2.** Forest plot for injection site reaction.

**Figure S3.** Forest plot for dyspepsia.

**Figure S4.** Forest plot for constipation.

**Figure S5**. Forest plot for pancreatitis.

**Figure S6**. Forest plot for cardiovascular death, MI, or stroke.

**Figure S7.** A Network graphs of for all safety outcomes: **(A)** any AE that emerged during treatment compared; **(B)** nausea; **(C)** diarrhea; **(D)** vomiting; **(E)** all-cause death; **(F)** severe hypoglycemia; **(G)** serious AE; **(H)** headache; **(I)** injection site reaction; **(K)** dyspepsia; **(L)** constipation; **(M)** AE leading to drug discontinuation; **(N)** cardiovascular death, MI, stroke or coronary revascularization.

.
**Figure S8.** Sensitivity analysis for all-cause death by excluding Nicholas et al. 2025

**Figure S9.** Sensitivity analysis for severe hypoglycemia by excluding Nicholas et al. 2025

**Figure S10.** Sensitivity analysis for serious AE by excluding Nicholas et al. 2025

**Figure S11.** Sensitivity analysis for AE leading to drug discontinuation by excluding Nicholas et al. 2025

**Figure S12.** Sensitivity analysis for any AE that emerged during treatment by excluding Nicholas et al. 2025

**Figure S13.** Sensitivity analysis for pancreatitis by excluding Nicholas et al. 2025

**Figure S14.** Sensitivity analysis for vomiting by excluding Nicholas et al. 2025

**Figure S15.** Sensitivity analysis for diarrhea by excluding Nicholas et al. 2025

**Figure S16.** Sensitivity analysis for nausea by excluding Nicholas et al. 2025

**Figures S17-S47.** Sensitivity Analyses Excluding Billings et al. 2025 or Inagaki et al. 2022

**Note: Figures S17-S47 are attached in Supplementary File Part III.**
