# Supplementary material for: Efficacy and Safety of Tirzepatide Versus Dulaglutide in Type 2 Diabetes With or Without Established Atherosclerotic Cardiovascular Disease: A Network Meta‐Analysis of Randomized Clinical Trials
Source: Endocrinol Diabetes Metab. 2026 Sep 7;9(5):e70313. doi: 10.1002/edm2.70313 (PMC13550879; doi:10.1002/edm2.70313)
Supplement: Supplementary file 3 [file EDM2-9-e70313-s003.docx]

Supplementary File Part III

**Title:** Efficacy and Safety of Tirzepatide Versus Dulaglutide in Type 2 Diabetes with or without Established Atherosclerotic Cardiovascular Disease: A Network Meta-Analysis of Randomized Clinical Trials

***Figures S17-S47.*** *Sensitivity Analyses Excluding Billings et al. 2025 (****SURPASS-SWITCH****) or Inagaki et al. 2022 (****SURPASS J-mono)***

# **(1) Sensitivity Analyses Excluding Billings et al. 2025 (SURPASS-SWITCH)**

**Figure S17.** Adverse Event Leading to Drug Discontinuation — Sensitivity Analysis Excluding Billings et al. 2025

**Figure S18.** All-Cause Death — Sensitivity Analysis Excluding Billings et al. 2025

**Figure S19.** Body Weight Change from Baseline at Week 12 — Sensitivity Analysis Excluding Billings et al. 2025

**Figure S20.** Body Weight Change from Baseline at Week 16 — Sensitivity Analysis Excluding Billings et al. 2025

**Figure S21.** Constipation — Sensitivity Analysis Excluding Billings et al. 2025

**Figure S22.** Diarrhea — Sensitivity Analysis Excluding Billings et al. 2025

**Figure S23.** Dyspepsia — Sensitivity Analysis Excluding Billings et al. 2025

**Figure S24.** Headache — Sensitivity Analysis Excluding Billings et al. 2025

**Figure S25.** Injection Site Reaction — Sensitivity Analysis Excluding Billings et al. 2025

**Figure S26.** Mean Change in HbA1c from Baseline to Week 12 — Sensitivity Analysis Excluding Billings et al. 2025

**Figure S27.** Mean Change in HbA1c from Baseline to Week 24 — Sensitivity Analysis Excluding Billings et al. 2025

**Figure S28.** Nausea — Sensitivity Analysis Excluding Billings et al. 2025

**Figure S29.** Pancreatitis — Sensitivity Analysis Excluding Billings et al. 2025

**Figure S30.** Serious Adverse Event — Sensitivity Analysis Excluding Billings et al. 2025

**Figure S31.** Severe Hypoglycemia — Sensitivity Analysis Excluding Billings et al. 2025

**Figure S32.** Vomiting — Sensitivity Analysis Excluding Billings et al. 2025

# **(2) Sensitivity Analyses Excluding Inagaki et al. 2022 (SURPASS J-mono)**

**Figure S33.** Any Treatment-Emergent Adverse Event — Sensitivity Analysis Excluding Inagaki et al. 2022

**Figure S34.** Body Weight Change from Baseline at Week 12 — Sensitivity Analysis Excluding Inagaki et al. 2022

**Figure S35.** Body Weight Change from Baseline at Week 16 — Sensitivity Analysis Excluding Inagaki et al. 2022

**Figure S36.** Constipation — Sensitivity Analysis Excluding Inagaki et al. 2022

**Figure S37.** Diarrhea — Sensitivity Analysis Excluding Inagaki et al. 2022

**Figure S38.** Dyspepsia — Sensitivity Analysis Excluding Inagaki et al. 2022

**Figure S39.** Headache — Sensitivity Analysis Excluding Inagaki et al. 2022

**Figure S40.** Injection Site Reaction — Sensitivity Analysis Excluding Inagaki et al. 2022

**Figure S41.** Mean Change in HbA1c from Baseline to Week 12 — Sensitivity Analysis Excluding Inagaki et al. 2022

**Figure S42.** Mean Change in HbA1c from Baseline to Week 24 — Sensitivity Analysis Excluding Inagaki et al. 2022

**Figure S43.** Nausea — Sensitivity Analysis Excluding Inagaki et al. 2022

**Figure S44.** Pancreatitis — Sensitivity Analysis Excluding Inagaki et al. 2022

**Figure S45.** Serious Adverse Event — Sensitivity Analysis Excluding Inagaki et al. 2022

**Figure S46.** Severe Hypoglycemia — Sensitivity Analysis Excluding Inagaki et al. 2022

**Figure S47.** Vomiting — Sensitivity Analysis Excluding Inagaki et al. 2022
